# The Brain Imaging for Global Health (BRIGHT) Project: Longitudinal cohort study protocol

**DOI:** 10.12688/gatesopenres.14795.1

**Published:** 2023-10-18

**Authors:** Sarah Lloyd-Fox, Sam McCann, Bosiljka Milosavljevic, Laura Katus, Anna Blasi, Chiara Bulgarelli, Maria Crespo-Llado, Giulia Ghillia, Tijan Fadera, Ebrima Mbye, Luke Mason, Fabakary Njai, Omar Njie, Marta Perapoch-Amado, Maria Rozhko, Fatima Sosseh, Mariama Saidykhan, Ebou Touray, Sophie E. Moore, Clare E. Elwell, The BRIGHT Project team

**Affiliations:** 1 Psychology, University of Cambridge, Cambridge, England, UK; 2 Psychological Sciences, Birkbeck University of London, London, England, UK; 3 Women's and Children's Health, Kings College London, London, UK; 4 School of Human Sciences, University of Greenwich, London, England, UK; 5 Medical Physics and Biomedical Engineering, University College London, London, England, UK; 6 Institute of Lifecourse and Medical Sciences, University of Liverpool, Liverpool, England, UK; 7 The Medical Research Council Unit The Gambia at the London School of Hygiene and Tropical Medicine, Banjul, The Gambia; 8 Psychology, University of East London, London, England, UK

**Keywords:** Gambia, UK, infancy, development, undernutrition, longitudinal, neuroimaging, global health, fNIRS, EEG

## Abstract

There is a scarcity of prospective longitudinal research targeted at early postnatal life which maps developmental pathways of early-stage processing and brain specialisation in the context of early adversity. Follow up from infancy into the one-five year age range is key, as it constitutes a critical gap between infant and early childhood studies. Availability of portable neuroimaging (functional near infrared spectroscopy (fNIRS) and electroencephalography (EEG)) has enabled access to rural settings increasing the diversity of our sampling and broadening developmental research to include previously underrepresented ethnic-racial and geographical groups in low- and middle- income countries (LMICs). The primary objective of the Brain Imaging for Global Health (BRIGHT) project was to establish brain function - using longitudinal data from mother - for-age reference curves infant dyads living in the UK and rural Gambia and investigate the association between context-associated moderators and developmental trajectories across the first two years of life in The Gambia. In total, 265 participating families were seen during pregnancy, at 7–14 days, 1-, 5-, 8-, 12-, 18- and 24-months post-partum. An additional visit is now underway at 3–5 years to assess pre-school outcomes. The majority of our Gambian cohort live in poverty, but while resource-poor in many factors they commonly experience a rich and beneficial family and caregiving context with multigenerational care and a close-knit supportive community. Understanding the impact of different factors at play in such an environment ( *i.e.*, detrimental undernutrition *versus* beneficial multigenerational family support) will (i) improve the representativeness of models of general cognitive developmental pathways from birth, (ii) identify causal pathways of altered trajectories associated with early adversity at both individual and group level, and (iii) identify the context-associated moderators ( *i.e.* social context) that protect development despite the presence of poverty-associated challenges. This will in turn contribute to the development of targeted interventions.

## Introduction

### Background

The first 1000 days of life, which describes the developmental period between conception and two years of age, is characterised by prodigious physiological, psychological and physical change. As such, this period represents a critical window for brain development, during which plasticity to environmental factors is greatest. According to UNICEF, 19.5% of the world’s children live in poverty, the majority of whom reside within sub-Saharan Africa (51.7%) and South Asia (35.7%). Infants and children growing up in poverty may be exposed to a range of biological and/or psychosocial risk factors both pre- and postnatally. Such risk factors include lower parental income and educational level, parental mental health issues, reduced access to recreational and educational activities (particularly in rural communities), undernutrition, food insecurity, environmental hazards and poor sanitation ( Giovanelli *et al*., 2016; Jensen *et al*., 2017; Smith *et al*., 2015; Worku *et al*., 2018). While resource poor in many factors, there can also be many beneficial context-associated moderators, for example some communities who grow up in poverty also experience a rich and beneficial family and caregiving context with multigenerational social support and a close-knit supportive community. The impact of environment on neurocognitive development is therefore dynamic and multi-faceted, affecting biological, social, and behavioural developmental processes. For example, undernourished infants may seek, and consequently receive, less stimulation from caregivers. This lack of social stimulation is, in turn, linked to changes in brain function which are likely to precede changes in behaviour ( East *et al*., 2017). Despite these multifaceted links between different factors, evidence examining the impact of poverty-related risk and developmental outcomes oftentimes focusses on only narrow subsets of relevant factors, which leaves open questions regarding the interplay across domains. As noted by Nobel Laureate Esther Duflo in 2019, ‘Our goal is to make sure that the fight against poverty is based on scientific evidence. It starts from the idea that often the poor are reduced to caricatures and often even people who try to help them do not actually understand what are the deep roots of the problems’ ( Cho, 2019). Here, we describe the rationale behind and cohort characteristics of the Brain Imaging for Global Health ( BRIGHT) project, which works with family cohorts in the UK and The Gambia. We first present relevant literature that informed the design of the BRIGHT project, before providing details on the study protocol and characteristics of the two cohorts.

Childhood poverty has been associated with lower performance on language, memory and cognitive control tasks ( Farah *et al*., 2006). At a global level, this is reflected by one third of pre-school-aged children in low-and-middle-income countries (LMICs) failing to reach age-appropriate milestones in cognitive and/or socio-emotional development ( McCoy *et al*., 2016). Within the first years of life, one major poverty-associated risk factor that poses a considerable risk to early child development is stunting (low length/height for age against an international reference), which occurs as the result of chronic growth failure and affects one in five children under five years of age globally ( Development Initiatives, 2018). Furthermore, the interplay between the impact of undernutrition and compensatory factors ( *i.e.*, positive parenting practices), thought to scaffold early child development, is complex. A recent study in rural Cambodia looking at the joint role of parenting and nutritional status – in relation to inequities in family wealth – found that, while more stimulating and supportive parenting practices were associated with improved developmental outcomes in three to five year olds, this was strongest for non-stunted children ( Berkes *et al*., 2019). We therefore urgently need to further our understanding of brain and cognitive development during early childhood in the context of poverty-associated risk factors. This is especially relevant as compromised development of a core set of age-appropriate skills in childhood has a significant impact on subsequent academic achievement, mental health and economic status - and consequently the potential to lead full and productive lives and support future generations ( Alderman *et al*., 2014; Hackman & Farah, 2009; Martorell *et al*., 2010; Victora *et al*., 2008). The United Nations Sustainable Development Goals have consequently identified the reduction of poor cognitive development during childhood in LMICs as a key priority for global health research and interventions ( UN, 2015). All LMICs fall within what are also known as Majority World countries, where 85% of the world’s population live ( Alam, 2008). However, over the past 15–30 years, only 3 – 17% of published child development journal articles ( Moriguchi, 2022; Nielsen *et al*., 2017) are from Majority World countries and only 5% of child development interventions ( Draper *et al*., 2023), meaning that the world’s child population are under-represented in our theoretically driven understanding of development ( Draper *et al*., 2022). In the following section we review some of the existing literature on links between poverty and brain development as well as describe how a small number of studies are beginning to address the under-representation of child development research in LMIC (Majority World) contexts.

Over the last decade several large-scale studies from the United States have shown links between poverty and brain development in childhood ( Barch *et al*., 2016; Hair *et al*., 2015; Luby *et al*., 2013; Noble *et al*., 2015); for example, children of parents with high school education had roughly 3% less cortical volume than those with university level education, and those with parental incomes below $25,000 had 6% less than those making over $150,000 ( Noble *et al*., 2015). These findings have led to models relating specific components of socioeconomic status (SES), stress and brain structural and functional development ( Farah, 2017; Hackman & Farah, 2009; Noble *et al*., 2012). However, the majority of these studies rely heavily on correlational analyses in later childhood rather than studying development at an early age to fully understand the mechanistic processes driving these differences (for a review of studies that have looked at general differences in brain volume and poverty in infancy see Hurt & Betancourt, 2016). While the development of new methodology has increased our understanding of brain and cognitive development in infants and young children over the last decade, this research has been largely restricted to financially mobile participants within high-income countries ( Henrich *et al*., 2010). Studies that examine the impact of extreme poverty (defined by the World Bank as living on less than $1.9 per household member per day) on brain development are extremely scarce. Furthermore, it remains unclear whether poverty-associated risk factors influence brain development more severely during sensitive periods of development ( *i.e.* fetal life/early infancy/early childhood), or whether their impact builds over time depending on the chronicity, pervasiveness, severity and/ number of incidences ( Berens *et al*., 2017; Jensen *et al*., 2017). Furthering our understanding of the developmental impact of early environmental adversity in both the short- and long-term is thus of high priority, particularly during the understudied period of the first 1000 days of life.

A further consideration is that the majority of child development research conducted in LMICs has been limited to the use of behavioural assessments of cognitive development to measure the effect of exposure to early adversity. Such measures are often undertaken later in childhood rather than at the time that vulnerability to exposure is most critical *i.e.* during prenatal and early postnatal life ( Sabanathan *et al*., 2015). Furthermore, it is important to note that developmental trajectories of perceptual, motor and language domains have different timescales and cascading effects on one another. Therefore, they may have different key periods of sensitivity to insults (for an example of a key sensitive period for visual cortex see Hensch, 2005). Thus, it is imperative that neurocognitive development is studied from as close to birth as possible – ideally with a longitudinal framework to track age-related changes – taking contemporaneous measurements of brain function and behaviour in parallel with measurements of exposure to environmental challenges. Furthermore, such research could offer new pathways for the provision of widely applicable, objective paradigms and methods that can assess early brain development in hard-to-reach populations ( Isaacs, 2013). This would be synergistic with current large scale global health initiatives to optimise behavioural measures of early cognitive development ( Murray-Kolb *et al*., 2014; Richter *et al*., 2019) such as the Global Scales of Early Development (GSED) ( Cavallera *et al*., 2023). Furthermore the introduction of objective brain imaging paradigms to global health research could address some of the current challenges associated with behavioural measures of child development ( Isaacs *et al*., 2008; Perkins *et al*., 2017). The optimisation of tools for measuring neurocognitive development will in turn support the development of early intervention strategies from the first days and months of life offering the potential for large lifetime cost savings ( *i.e.* “1001 Critical Days” cross party manifesto, UK; Sure Start ( Cattan *et al*., 2021)).

Over the last five to ten years, several research collaborations have been established to bring new neurocognitive technology ( *i.e.* eye-tracking and neuroimaging tools) to the field of infant and early child brain and cognitive development research in LMICs: these include within The Gambia ( Begus *et al*., 2016; Katus *et al*., 2019; Katus *et al*., 2020; Katus *et al*., 2023; Katus *et al*., 2022b; Lloyd-Fox *et al*., 2014; Lloyd-Fox *et al*., 2017; Lloyd-Fox *et al*., 2019); Cote D’Ivoire ( Jasinska & Guei, 2018); Guinea Bissau ( Roberts *et al*., 2017); Malawi ( Forssman *et al*., 2017; Pyykkö *et al*., 2019; Pyykkö *et al*., 2020); Bangladesh ( Jensen *et al*., 2019; Perdue *et al*., 2019; Turesky *et al*., 2019; Xie *et al*., 2018); India ( Wijeakumar *et al*., 2019); South Africa ( Wedderburn *et al*., 2020) and Brazil ( Alarcão *et al*., 2021). These global infant and child brain development studies cover age ranges from 0 – 65 months of age, reporting on areas as wide ranging as social information processing ( Lloyd-Fox *et al*., 2017; Perdue *et al*., 2019; Xie *et al*., 2019), early brain anatomical and connectivity development ( Collins-Jones *et al*., 2021; Fishell *et al*., 2020; Turesky *et al*., 2020; Turesky *et al*., 2019), the development of brain networks associated with visual working memory ( Wijeakumar *et al*., 2019) and the development of attentional/neural markers of habituation and novelty detection ( Katus *et al*., 2020; Katus *et al*., 2023; Katus *et al*., 2022b; Lloyd-Fox *et al*., 2019). Finally, a recent step change in research has been to begin to use measures of brain and cognitive development to understand the impact of interventions within global health studies; as evidenced by the recent work by Alarcão and colleagues in Brazil to measure the efficacy of a home-visiting program for adolescent mothers for enhancing early infant brain development and behaviour ( Alarcão *et al*., 2021).

In this paper we describe the BRIGHT project, a follow-on from a pilot study, which ran from 2012–2014, in which we demonstrated the feasibility of combined neuroimaging, behavioural assessments and growth measures in longitudinal and cross-sectional studies from birth to 24 months of age in rural Gambia ( Begus *et al*., 2016; Lloyd-Fox *et al*., 2017; Lloyd-Fox *et al*., 2014; Papademetriou *et al*., 2013). Importantly we showed that fNIRS, can be easily implemented in rural contexts such as in The Gambia and used from the first weeks of life to provide quantitative and objective markers of neurocognitive function. We identified testing paradigms that elicit reliable brain responses that can be used to chart development as a function of age, and which aligned with findings in age-matched groups of infants from studies conducted in the UK. As part of our pilot study, we also successfully performed a quality control assessment of the adaptation and administration of a behavioural assessment, the Mullen Scales of Early Learning (MSEL), for use in rural Gambia ( Milosavljevic *et al*., 2019). The BRIGHT project has been established to extend this pilot phase to a larger longitudinal observation cohort study of infant and early child development from birth to two years of age, with an additional follow-up at pre-school age (3–5 years).

### The BRIGHT project design overview

The BRIGHT project (Phase I), which ran from 2015 – 2020, established two prospective cohorts of families in the UK and The Gambia using a longitudinal multi-methods approach. Families were recruited during pregnancy and, following delivery, longitudinal measures of infant brain and cognitive development were conducted from 0–24 months of age across 10 data collection phases: antenatal – recruitment and 32–36 weeks’ gestation, and postnatal – 1–3, 7–14 days, 1, 5, 8, 12, 18 and 24 months of age. Additional data on diet and health were collected continuously across this time period. The project implemented brain imaging measures (fNIRS and electroencephalography [EEG]), neurocognitive behavioural measures (utilising eye-tracking methods), population-specific cognitive developmental measures (MSEL and the Communicative Development Inventory [CDI]), family-caregiving assessments (Family Care Indicators [FCI]), caregiver-infant interaction videos and questionnaires) and home environment measures (Language Environment Analysis [LENA]) alongside regular collection of biological, socioeconomic, parental health and nutritional measures at both sites. This data collection framework was implemented to allow the modelling of longitudinal changes in brain function, cognitive development, and growth within the rural Gambian population. Further, the collection of parallel behavioural and environmental data was designed to enable the identification of critical developmental moderators, mediators, and markers of risk and resilience. The purpose of the BRIGHT project is to firstly establish longitudinal trajectories across populations, and secondly to provide a framework for in-depth investigations of inter-individual differences within the Gambian cohort. Given that neuroimaging data provided the backbone of this project, it was essential that a UK cohort was also established to measure the longitudinal developmental trajectories of the different fNIRS and EEG paradigms across different populations as several of these had not been studied across this longitudinal time span before in any population internationally. This was chosen to broadly match the context of previously acquired developmental neuroimaging data, given that to date the vast majority of research of this kind has been undertaken in high income countries (HICs).

The target cohort sizes (The Gambia n=200, UK n=60,) were based on previous infant fNIRS and EEG studies conducted in the UK, which indicated that sample sizes from 20 (moderate effect size) to 42 (small effect size) were sufficient to determine regions of significant cortical brain activation in response to stimuli. The Gambian cohort was designed to be larger to allow within-cohort sub-group comparisons and individual differences analyses; for example, grouping by growth trajectories (mild, medium and severe markers of undernutrition) on the assumption that approximately 25–30% of the cohort would be stunted (z-score of length-for-age < 2 standard deviations below the WHO reference) by two years of age ( Nabwera *et al*., 2017).

### BRIGHT Kids (BRIGHT project phase II)

Previous research highlights a marked impact of exposure to early adversity and neurobehavioral outcomes at preschool age. With this in mind, in 2023 we conducted a follow-up assessment at preschool age in the Gambian cohort of the BRIGHT project at 3–5 years. This cross-sectional follow up will allow us to examine additional questions, regarding the long-term stability of our infant neural markers to predict long-term outcomes.

### Objectives

The primary objectives of the BRIGHT project are to:

(1) develop brain and neurocognitive function-for-age curves from birth to 24 months of age using prospective longitudinal datasets from the UK and The Gambia. These reference curves will be used to enable age-adjusted group comparisons of differences in average trajectories, group-wise differences in variability, and for characterizing the range of individual developmental trajectories within each cohort.
(2) establish the association between context-associated moderators, including poverty-associated risk factors ( *i.e.*, undernutrition and consequent growth faltering), and developmental trajectories across the first two years of life in The Gambia.
(3) establish the association between context-associated moderators and developmental trajectories across the first two years of life in The Gambia and pre-school outcomes at three to five years.

Secondary aims of interest are to:

1. Assess whether infants with similar trajectories of growth have the potential to reach the same developmental milestones within the first 24 months of life.
2. Establish whether neuroimaging markers of brain function are more robust indicators of development within individual infants across age, as compared to behavioural measures.
3. Assess the capability of fNIRS, EEG and eye-tracking methods to deliver specific and early biomarkers of altered developmental pathways.

Here we describe the formation of a common BRIGHT study protocol across the two sites (The Gambia and the UK) and, where appropriate, site-specific additional measures, particularly those focused on family context, nutrition, diet, and biological samples are presented for The Gambia. We briefly outline how we recruited participants at each site; selected and implemented experimental neurocognitive and behavioural measures at each site; how we standardised lab practices across sites to ensure comparability; and how we developed analytical pipelines for the different datasets. We also describe the demographic and socioeconomic distribution of our cohorts.

## Methods

### The Gambian site and population

The Gambia is situated on the West coast of Africa, bordering Senegal. The majority (60%) of the roughly 2.4 million inhabitants of The Gambia live in the coastal regions surrounding the capital, Banjul, while the remainder of the population live rurally, often supporting themselves through subsistence farming ( Hennig *et al*., 2017) living in extended, multi-generational households ( Brotherton *et al*., 2021; Kea, 2013; Sear & Mace, 2009). The Gambia is one of the lowest ranking countries with regard to gross national income, years of schooling, and life expectancy, with over half of adults never having received formal education ( Hennig *et al*., 2017). School attendance has risen rapidly over the last decades thanks to the introduction of free universal education, and 97% of children now attend to primary level relative to 66.7% in the early 1970s ( The Gambia Government National Education Statistics, 2018; CEICdata.com). Preschool education has also become increasingly available across the timeframe of the BRIGHT project ( Blimpo *et al*., 2019). Childcare is viewed as a shared responsibility among family members, with grandmothers and older sisters having the biggest role in supporting parents ( Brotherton *et al*., 2021; Sear & Mace, 2009). Islam is the predominant religion and raising children in accordance with religious and community values is of high importance ( Sosseh *et al*., 2023). Marriages are commonly polygamous with over half of married women living with one or two co-wives ( Hennig *et al*., 2017), though gendered hierarchies and intra-household relations are dynamic and subject to change ( Kea, 2013; Kea, 2020). Furthermore, over the past decades infant and child mortality has decreased, birth spacing has increased, and overall family size has reduced ( Nabwera *et al*., 2017).

The Gambian arm of the BRIGHT project was hosted at a rural site of the Medical Research Council The Gambia Unit at the London School of Hygiene and Tropical Medicine (MRCG@LSHTM; www.mrc.gm). The UK Medical Research Council (MRC) has a long-standing research partnership with The Gambia, established in the late 1940’s. Currently, research conducted within MRCG@LSHTM is focused on three broad themes centred around major public health priorities, specifically Vaccines and Immunity, Disease Control and Elimination, and Nutrition and Planetary Health; the latter of which the BRIGHT project is situated within.

The BRIGHT project was undertaken at the Keneba Field Station of MRCG@LSHTM, situated in the rural West Kiang region, 145 km inland from the capital. Seasonality has an impact on nutrient availability for the population living here as weather patterns alternate between four months of heavy rainfall (July–October) and eight months of extreme dryness - directly affecting the availability of key nutrients ( Moore *et al*., 1997). In 2015 at the onset of the project, the Keneba Field Station was relatively isolated, accessed via unmade roads and required to independently maintain all facilities necessary for research and clinical care ( *e.g.*, generator powered electricity, bore hole water supply, satellite communication). However, over the course of the study, the country and local region have been witnesses to several changes. At the local level several infrastructure improvements have been made, including the road being tarmacked, and therefore allowing greater access to urban resources, and the field station and local community now have nationally sourced electrical power. BRIGHT project participants were drawn from Keneba and surrounding villages within a 20km radius of the field station.

**Figure 1.**
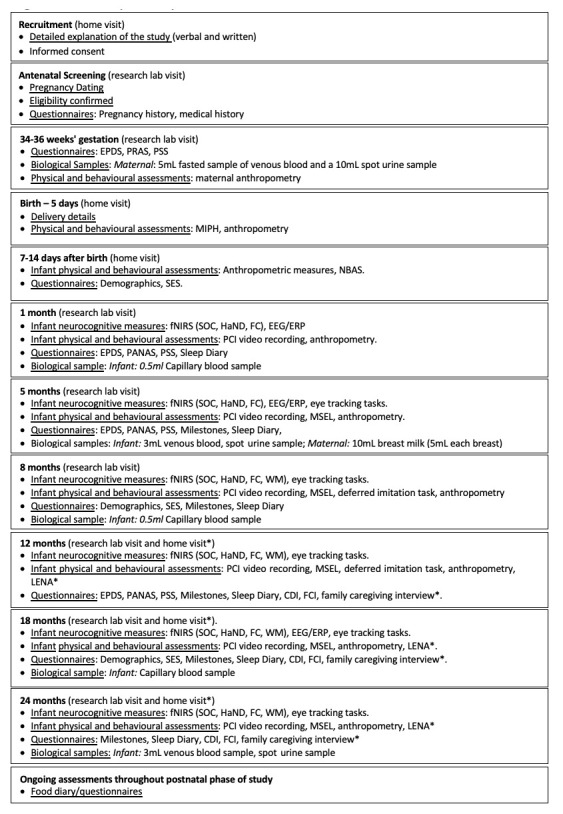
Summary of study visits: The Gambia. EPDS, Edinburgh Postnatal Depression Scale; PRAS, Pregnancy-related Anxiety Scale; PSS, Perceived Stress Scale; PANAS, Positive and Negative Affect Scale; NBAS, Neonatal Behavioural Assessment Scale; SES, Socioeconomic Status; MIPH, Maternal and Infant Physical Health; fNIRS, functional near infrared spectroscopy; EEG/ERP, electroencephalography/event related potentials; SOC, Social versus Non-Social Response; HaND, Habituation and Novelty Detection; FC, Functional Connectivity Networks; WM, Working Memory; DI, Deferred Imitation task; PCI, Parent-Child Interaction; MSEL, Mullen Scales of Early Learning; LENA, Language Environment Analysis; FCI, Family Care Indicators; CDI, Communicative Development Inventory; * indicates assessments undertaken in the family’s home at the later time points.

All women of reproductive age (18–45 years) who were reported to be pregnant within the West Kiang Demographic Surveillance System between June 2016 and March 2018, spoke Mandinka as their primary language, and were expected to reside in West Kiang for the duration of the project were invited to participate (see Figure 1 for recruitment pathway in The Gambia). Further eligibility criteria for study participation pertaining to the pregnant women included: carrying a singleton pregnancy, < 36 weeks’ gestation on presentation to the first antenatal study visit and being medically fit to participate, as determined by the study midwife. The project was designed to recruit participants so that deliveries were spread evenly throughout the recruitment period, aiming for around 10–15 deliveries per month. This was to ensure that workload was achievable and as consistent as possible, ensuring timely scheduling of follow-up visits. For this reason, an additional exclusion criterion of ‘gestational age incompatible with study requirements’ was introduced. From an ultrasound scan at the first antenatal study visit, gestational age was measured, and expected delivery date calculated. If a participant was due to deliver in a month that was already at full capacity, they were excluded at this point. Postnatally, mother-infant dyads were excluded from the project if the infant was diagnosed with a developmental disability *e.g.*, Down’s Syndrome or cerebral palsy. Participants were free to withdraw from participation at any point in the study.

### The UK site and population

**Figure 2.**
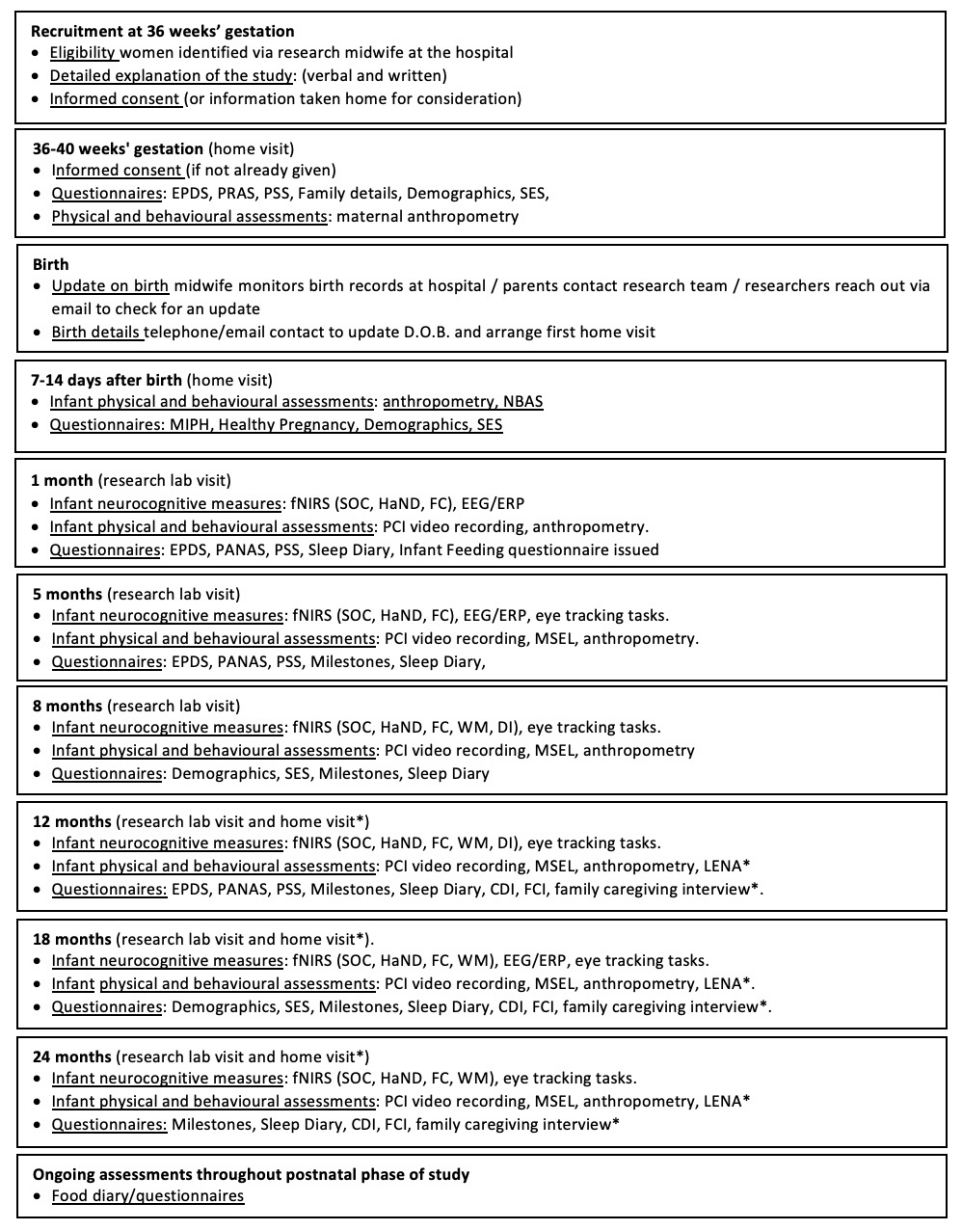
Summary of study visits: The UK. Abbreviations: EPDS, Edinburgh Postnatal Depression Scale; PRAS, Pregnancy-related Anxiety Scale; PSS, Perceived Stress Scale; PANAS, Positive and Negative Affect Scale; NBAS, Neonatal Behavioural Assessment Scale; SES, Socioeconomic Status; MIPH, Maternal and Infant Physical Health; fNIRS, functional near infrared spectroscopy; EEG/ERP, electroencephalography/event related potentials; SOC, Social versus Non-Social Response; HaND, Habituation and Novelty Detection; FC, Functional Connectivity Networks; WM, Working Memory; DI, Deferred Imitation task; PCI, Parent-Child Interaction; MSEL, Mullen Scales of Early Learning; LENA, Language Environment Analysis; FCI, Family Care Indicators; CDI, Communicative Development Inventory; * indicates assessments undertaken in the family’s home at the later time points.

In the UK, participants were recruited from the city of Cambridge and surrounding villages. Demographically, the population in Cambridgeshire is representative of that across the UK with regard to ethnicity, employment rates and family structure ( Cambridge County Council, 2011). The area however differs from the rest of the UK with regard to levels of education within the population, with twice as many inhabitants holding a higher education degree ( Cambridge County Council, 2011). The research involved in the UK arm of the BRIGHT project was conducted at dedicated facilities either within the Evelyne Perinatal Imaging Unit at the Rosie Hospital, Cambridge University Hospitals NHS Foundation Trust or within the Department of Psychology, University of Cambridge. Once per week during the recruitment phase, families who attended an antenatal clinic at the Rosie Maternity Unit at Cambridge University Hospitals between June 2016 and January 2017, with a healthy singleton pregnancy less than 36 weeks gestational age, were approached and given information about the project (see Figure 2 for UK recruitment pathway).

### Ethical considerations

Protocols were approved by the relevant committee at each site. In The Gambia, ethical approval was given by the joint Gambia Government - MRC Ethics Committee (SCC 1351) and the Scientific Coordinating Committee at the MRC Unit The Gambia. Additional approval was granted for the BRIGHT Kids follow up (Project reference 22737). Informed consent was obtained in writing, or via thumbprint if individuals were unable to write, from all parents/carers prior to participation. In the UK, the study was approved by the National Research Ethics Service East of England Committee, NHS Health Research Authority (REC reference 13/EE/0200), and informed written consent was obtained from parents of infants to participate. The project is guided by a consistent set of principles which ensured that the infants’ and child’s wellbeing is always prioritised. Infants/children are always with their caregiver. The protocols were designed to be engaging and interesting to the infants and children, and the setup comfortable. Caregivers were made aware that the study can be interrupted, rescheduled, or stopped at any time if the infant/child became fussy or tired, or, if the caregiver expressed a wish to end the study. Each research team includes researchers fluent in Mandinka and/or English, as relevant. Data protection and confidentiality shape our approach to data sharing within the BRIGHT research team and externally, and is detailed in the *Standardisation of protocol across sites* section.

### Study protocol

The full study protocol is shown in Table 1 and outlined below. The draft protocol was developed through the integration of expertise from our international multi-disciplinary (psychology, neuroscience, medical physics and bioengineering, maternal and infant health and nutrition, global health) research leadership team. Following this, all measures were reviewed during several multi-site web-based meetings to identify the necessary adaptations and translations required to ensure each paradigm and assessment was appropriate for the population and culture of the cohort. Furthermore, some field testing and adaptation had already been undertaken in previous pilot phases within our research group ( Lloyd-Fox *et al*., 2014; Milosavljevic *et al*., 2019).

**Table 1.** Summary of BRIGHT protocol. Note on abbreviations: * The UK only; ▵ The Gambia only; ^ Recorded every two weeks from 2 weeks to 24 months of age; L,W, H, HC,MUAC, KHL (L – length, W – weight, H – height, HC – head circumference, MUAC – mid to upper arm circumference, KHL – knee to heel length).

Study Measure Time point:	Antenatal	Birth	7–14 d	1 mo	5 mo	8 mo	12 mo	18 mo	24 mo
Neuroimaging measures									
fNIRS: Social/Non-social				**x**	**x**	**x**	**x**	**x**	**x**
fNIRS: Habituation and Novelty Detection				**x**	**x**	**x**	**x**	**x**	**x**
fNIRS: Functional connectivity				**x**	**x**	**x**	**x**	**x**	**x**
fNIRS: Working memory						**x**	**x**	**x**	**x**
fNIRS */Behavioural: Deferred imitation						**x**	**x**		
EEG: Auditory Oddball				**x**	**x**			**x**	
Behavioural/Neurocognitive measures									
Neonatal Behavioural Assessment Scale (NBAS)			**x**						
Eye-tracking: Cognitive control					**x**	**x**	**x**	**x**	**x**
Eye-tracking: Habituation					**x**	**x**	**x**		
Eye-tracking: Gap/Overlap					**x**	**x**	**x**	**x**	**x**
Eye-tracking: Non-social contingency					**x**	**x**	**x**		
Eye-tracking: Face popout					**x**	**x**	**x**	**x**	**x**
Eye-tracking: Dynamic scenes					**x**	**x**	**x**	**x**	**x**
Eye-tracking: Word-picture-matching								**x**	**x**
Mullen Scales of Early Learning (MSEL)					**x**	**x**	**x**	**x**	**x**
Parent-Child Interaction				**x**	**x**	**x**	**x**	**x**	**x**
LENA language assessment in home							**x**	**x**	**x**
LENA in PCI				**x ▵**	**x ▵**	**x ▵**	**x ▵**	**x**	**x**
Tablet-based Cognitive Assessment *								x *	x *
Questionnaires/Interviews – Infant/Child									
Communication Development Inventory (CDI)							**x**	**x**	**x**
Adapted Oxford Sleep Diary				**x**	**x**	**x**	**x**	**x**	**x**
Food Frequency Q (FFQ) ▵ ^		**x**	**x**	**x**	**x**	**x**	**x**	**x**	**x**
Infant feeding online Q (IFQ) * ^				**x**	**x**				
Food diary (Intake24UK) *						**x**	**x**	**x**	**x**
Early Childhood Development Index ^									
Questionnaires/Interviews – Family									
Edinburgh Postnatal Depression Scale (EDPS) – Maternal	**x**			**x**	**x**		**x**		**x**
Edinburgh Postnatal Depression Scale (EDPS) – Paternal *	**x**			**x**	**x**		**x**		**x**
Pregnancy Related Anxiety form (PRAS) – Maternal	**x**								
Pregnancy Related Anxiety form (PRAS) – Paternal *	**x**								
Pregnancy Specific Anxiety (PSA) – Maternal	**x**								
Pregnancy Specific Anxiety (PSA) – Paternal *	**x**								
Positive Negative Affect Schedule (PANAS) – Maternal				**x**	**x**		**x**		**x**
Positive Negative Affect Schedule (PANAS) – Paternal *				**x**	**x**		**x**		**x**
Perceived Stress Scale (PSS) – Maternal	**x**			**x**			**x**		**x**
Perceived Stress Scale (PSS) – Paternal *	**x**			**x**			**x**		**x**
Socioeconomic Status (SES)	x *		**x ▵**			x *		**x**	
Demographic and Family Information	x *		**x ▵**			x *		**x**	x *
Family details (from DSS – Gambia or antenatal call UK)	**x**					x *			x *
Family Caregiving Questionnaire (FCQ)							**x**	**x**	**x**
Family Care Indicators (FCI) ▵							**x**	**x**	**x**
Clinical measures / Medical details									
Healthy Pregnancy Questionnaire *			**x**						
Antenatal Medical form *	**x**								
Delivery Information and Baby check ▵		**x**							
Anthropometric measures – infant (L,W,HC,MUAC, KHL)		**x** (L,W,HC)	**x** (L,W,HC)	**x**	**x**	**x**	**x**	**x**	**x**
Anthropometric measures – mother (W, H)									
Maternal blood sample ▵	**x**								
Maternal urine sample ▵	**x**								
Maternal breast milk sample ▵					**x**				
Infant blood sample ▵				**x**	**x**	**x**	**x**	**x**	**x**
Infant urine sample ▵					**x**		**x**		**x**

During the protocol development phase, for paradigms that used images, videos, or audio that included people, actors representative of the ethnicity and language of the participants were used. For paradigms that included toys or objects (either real or in image form), appropriate representatives of the contextual environment of each cohort were identified. For The Gambia only, when appropriate, questionnaires were translated and administered in Mandinka ( *e.g.*, all mental health questionnaires, and the Mullen Scales of Early Learning [MSEL]). A full adaptation process with forward and backward translation by the authors and the BRIGHT Project team in Keneba was undertaken for all questionnaires and assessments (for an example see Milosavljevic *et al*., 2019). As Mandinka is not a written language and literacy rates among caregivers were low, the questionnaires were converted into interview versions and conducted by trained field assistants. Of note, where translations were undertaken for standardised measures that were not open-source and free to use ( *i.e.*, Mullen Scales of Early Learning [MSEL]) we purchased the equivalent number of copies of the original forms from the publisher that would be required to administer the measure at each age point. Neurocognitive measures were selected, where possible, on the basis of test re-test reliability and previous evidence indicating that they showed robust data quality ( *i.e.*, fNIRS ( Blasi *et al*., 2014); Eye-tracking ( Jones *et al*., 2019); EEG ( Dzhelyova *et al*., 2019; Räikkönen *et al*., 2003)). Within each battery of measures, we selected a combination of well tested and robust paradigms, and, when necessary to allow us to target particular cognitive domains or informative metrics, paradigms with novel designs were developed by the BRIGHT research group ( *i.e.*, fNIRS tasks to assess working memory, delayed imitation, habituation, repetition suppression and novelty detection). Tasks were administered in a pre-determined order across the study visit where possible ( *i.e.*, anthropometrics were taken at the end of the session to optimise infants’ attention and energy for experimental tasks), and also within a testing modality ( *i.e.*, in the fNIRS session infants viewed paradigms in a set order according to the stimulus presentation scripting framework). On occasion, when infants tired before completing the full session, families were invited to return on a separate day to complete the tasks, but where possible infants were encouraged to continue after a nap and/or feed within the same visit. We found that a second visit was required more often in The Gambian cohort than in the UK. Details of the session and completion of tasks were recorded in a Session Log Form at each visit.

***Neuroimaging measures***

**Electroencephalography (EEG)**

Electroencephalography (EEG) has a long-standing tradition in neurodevelopmental research. It provides a direct measure of infants’ neural responses to stimuli without requiring them to overtly respond or to follow task instructions. Through the use of innovative, wireless EEG hardware, it is now possible to implement EEG tasks in remote rural contexts and in the absence of standardised lab settings ( Katus *et al*., 2019). The EEG task implemented in the BRIGHT project assessed auditory habituation and novelty detection, at 1, 5 and 18 months of infant age (for a description of the full protocol see Katus *et al*., 2020). Due to the nature of the sounds (pure tones, bursts of white noise etc.), no adaptations had to be undertaken allowing for identical protocols at both project sites. Infants were presented with auditory stimuli for approximately 15 minutes while asleep (at 1 month) or awake (at 5 and 18 months): during the latter an experimenter quietly entertained the infant with bubbles or silent toys to maintain calm attention during the task.

**Functional near infrared spectroscopy (fNIRS)**

Functional near infrared spectroscopy (fNIRS) is a relatively recent addition to the battery of neuroimaging measures available to neurodevelopmental research. fNIRS measures the haemodynamic response to the neural activation measured by EEG. It has become the technique of choice for many studies given its ease of use with infants and young children, improved spatial resolution (relative to EEG) and low cost (relative to MRI) ( Gervain *et al*., 2023; Lloyd-Fox *et al*., 2010). In addition, fNIRS is relatively portable, opening a pathway for implementation in the remote and/or out-of-lab settings often associated with global health research contexts ( Blasi *et al*., 2019; Katus *et al*., 2019).

The fNIRS paradigms implemented in the BRIGHT project assessed a range of cognitive functions and domains, namely social cognition ( Lloyd-Fox *et al*., 2017), habituation and novelty detection ( Lloyd-Fox *et al*., 2019), working memory, deferred imitation and functional connectivity. Paradigms were included, at age-appropriate time points, across the 1, 5, 8, 12, 18 and 24 months, as well as in BRIGHT Kids at 3–5 years of age. Paradigms contained auditory and/or visual stimuli and were presented while infants were asleep at 1 month of age and while infants were awake and alert at all other time points. Audio and visual stimuli were adapted with site-relevant content (see the *Preliminary Results* section and Katus *et al*., 2019). The full fNIRS battery lasted 24 min for the shortest sessions at 1 and 5 months to up to 35 min for the longest at 8 and 12 months (where we included a live behavioural Deferred Imitation task). When accounting for preparation time such as settling the infant, taking head measurements, capping, and photographing headgear, the total assessment time was approximately 45 minutes. This multi-domain battery was designed to interrogate whether global health risk factors impact on development to result in global/cross-domain differences in brain activity or localized/domain-specific differences or altered function.

***Behavioural/neurocognitive assessments***

**Neonatal Behavioural Assessment Scale (NBAS)**

The NBAS is a structured clinical assessment of infant neurology and behaviour, which can be performed within the first few days of life. The NBAS is regarded as the most comprehensive examination of newborn behaviour available. It has been used across multiple cultures and in different LMICs (Zambia: Brazelton *et al*., 1976, Chile: Ayala *et al*., 2021, Mexico: Soler-Limón *et al*., 2019, Kenya: Super & Harkness, 2020). The NBAS is a standard protocol which requires an initial training period culminating in assessment and certification as described in *The Neonatal Behavioral Assessment Scale* Manual ( Brazelton & Nugent, 1995). Prior to the BRIGHT Project, we conducted a qualitative pilot study to assess the cultural acceptability and feasibility of using the NBAS within the rural low resource settings of families living in the West Kiang region of The Gambia. To this end, fifteen infants were assessed with the NBAS, and their families’ feedback showed that the NBAS was acceptable to parents in this population ( Bartram, 2018). In line with feedback from parents from other countries and populations, a few aspects of the assessment (specifically, shining a light over closed eyes while sleeping, covering eyes with cloth while awake and undressing the infant) were questioned or perceived negatively. While no items were altered or removed from the assessment, the more controversial items were introduced with special care during all study visits. In both the UK and The Gambia, the NBAS was performed between 7–14 days after birth during a pre-arranged home visit. Administration times for the NBAS ranged from 20 to 45 minutes, depending on the infants’ state of alertness.

**Eye-tracking neurocognitive battery**

Eye-tracking is a non-invasive and well-tolerated measure in infant neurodevelopmental research ( Jones *et al*., 2019). The tasks in our eye-tracking battery were selected to provide broad coverage of several key domains of neurocognitive functioning, including working memory ( Elsabbagh *et al*., 2009; Elsabbagh *et al*., 2013a; Johnson, 1995; Scerif *et al*., 2005), visual attention ( Kaldy *et al*., 2011), habituation ( Webb *et al*., 2010), reversal learning ( Wass, 2015), social *versus* non-social visual preference ( Elsabbagh *et al*., 2013b) and language learning ( Fernald *et al*., 2008). Table 1 displays the specific tasks used per age point. Many of the tasks, and all of the fixation stimuli that preceded a task, were gaze-contingent, that is they rely on the child’s gaze to proceed through the battery. The duration of the battery therefore varied slightly between participants but averaged 20 minutes.

The majority of the tasks focused on the use of visual stimuli (accompanied by simple alerting sounds) with no language modification required for use in The Gambia. Therefore, the eye-tracking battery was run with an existing stimulus package used in other longitudinal large scale cohort studies (developed by researchers at the Centre for Brain and Cognitive Development, Birkbeck, University of London) to aid future comparative analyses. An additional task (word-picture matching task) was developed specifically for the BRIGHT project to assess language comprehension of participants at the ages of 18 and 24 months. The task measured processing efficiency (speed and accuracy) in terms of infants’ ability to direct their gaze to one of two visual stimuli to match to a spoken target noun. This task was adapted from the behavioural Looking-while-Listening eye movement methodology used by Fernald and colleagues (2008), which had been recently adapted for a research project conducted in neighbouring Senegal ( Weber *et al*., 2017). During the design phase of the paradigm, common items were photographed and audio recordings of sentences relating to the photographed items were made in the appropriate language at each site. A series of pilot studies were then completed in both the UK and The Gambia to identify population- and age- appropriate word-picture stimulus pairs.

**Mullen Scales of Early Learning (MSEL)**

The MSEL measures cognitive ability and motor development using five scales: Gross Motor, Visual Reception, Fine Motor, Expressive Language, and Receptive Language. In both the UK and The Gambia, the MSEL was performed at visits to the research lab conducted at 5, 8, 12, 18 and 24 months of age, as well as at 3–5 years of age in The Gambia. During each visit, the MSEL was conducted using the standardized protocol appropriate for the age of the participant, as detailed in the Mullen Scales of Early Learning Manual and the Item Administration Book ( Mullen, 1995) and the MSEL training DVD. During a pilot phase to optimize and adapt the MSEL for use in The Gambia, n = 171 infants were tested across the age ranges described above ( Milosavljevic *et al*., 2019). The MSEL is broadly similar to the Bayley Scales of Infant and Toddler Development, both of which have been adapted for use across multiple countries, but originate in the U.S.A. and are normed to this population. A further restriction of this measure is that it is not open source and must be purchased, both the toolkit, manual and the assessment forms, *i.e.* a fee per participant is required to be paid.

**Parent-child interaction videos**

We assessed parent-child interaction styles in both the UK and The Gambia at 1, 5, 8, 12, 18 and 24 months of age. The parent and child engaged in a video recorded, 10-minute free-play session, which consisted of five minutes of play without toys and five minutes with a standardised collection of toys provided by the research team. The parent and child were seated on a mat in front of a mirror, to ensure that both of their faces were visible on the recording. At the younger ages, the infants were placed on a baby mat facing the parent, but, as they became more mobile, they were allowed to move around the room. The parent was instructed to play with their child as they normally would at home. For the younger time points the parent-infant dyad were left alone in the room to encourage a more relaxed environment, however, at later time points the researcher remained in the room to be able to move the camera around as the child became more mobile. These videos can be coded to assess multiple aspects of parental and child behaviour and engagement. Parental interactive characteristics have been shown to associate with child neural and cognitive development across a range of cultural contexts ( *i.e.* Bozicevic *et al*., 2016; Sethna *et al*., 2017).

**Language Environment Analysis system (LENA)**

The Language Environment Analysis system (LENA) provides automated counts of the linguistic environment. A digital language processor (DLP) is worn by the participant in the front pocket of a specially designed vest and able to record the audio environment within 1- to 3- metres. In BRIGHT, this was used to record the acoustic environment of the participants during a typical day in the home. Our standard protocol assessed the auditory home environment using LENA at the 12, 18 and 24 months of age. The recordings took place during two consecutive days, with seven hours of recording per day. During these recordings, parents were asked to complete a *Family Caregiving Questionnaire* or *Interview*. In the UK this was a logbook describing the main activities performed by the toddler, the locations in which the recording had taken place, who was around during the recording as well as technical details regarding the usage of the device. In The Gambia, this was conducted as an interview by a field assistant at the end of each recording day.

Via the LENA software, we extracted, 1) Adult word counts (AWC) defined as the number of adult words the key child hears - these adult words may be or may not be directed to the key child; 2) Key child vocalization counts (CVC), defined as words or prelinguistic babbling produced by the key child (crying and laughing sounds are not included in this category); 3) Conversational turn taking (CTT), identified as those instances when the key child and an adult speak one at a time in alternating turns. The reliability of these estimates has been shown in multiple languages; including English ( Gilkerson & Richards, 2008; Xu *et al*., 2009), French ( Canault *et al*., 2016), Dutch ( Busch *et al*., 2018), Spanish ( Weisleder & Fernald, 2013), Vietnamese ( Ganek & Eriks-Brophy, 2018) and Korean ( Pae *et al*., 2016). In line with previous research, field assistants, with Mandinka as their first language, transcribed a subset of recordings to determine reliability estimates for the Mandinka language. In addition to the home visits, within The Gambia a sub-sample (N = 40) of infants were followed more intensively from 1– 24 months of age with LENA recording included during the PCI sessions where the context is restricted to one parent and the target child.

***Tablet-basedased cognitive assessment***

The Babyscreen software application V1.83 (Hello Games, Guilford, UK) was used to measure key domains of neurodevelopment, including selective attention, working memory, and general learning ability. The task consists of 18 items and provides two performance variables: number of items accurately completed and speed of item completion ( Twomey *et al*., 2018). The task was administered on an Apple iPad (6 ^th^ generation, 9.7” screen). Given the young age of the BRIGHT participants and varying exposure to touch screen technology, two free play tasks were administered at the start of the testing session to familiarise the participants with the tablet, these involved drawing on the screen and moving shapes around.

In the UK, the task was administered to participants at 18 and 24 months of age ( Macrae *et al*., 2022). In The Gambia, several challenges arose, and the task was removed from the protocol: whereas pilots of the task were well-received by infants and parents, the density of assessment during the study visit meant that infants often were too fatigued to complete this task. Additionally, participants were more reluctant to touch or play with the tablet in the lab setting, even after encouragement from examiners and mothers. Attempts were made to complete the task at separate home visits. However, this had disadvantages as it increased the burden on the testing team and introduced wider variance in testing conditions. Therefore, the task was excluded from the main BRIGHT protocol in The Gambia.

***Questionnaires/interviews – infant/child***

**Communicative development inventory (CDI)**

The McArthur-Bates CDI ( Fenson *et al.*, 2007) was used to assess language development at the 12, 18 and 24 month time points. In the UK, the full English version was used, which consists of a vocabulary checklist that asks parents to report how many words their child can understand and how many they can understand and say. The questionnaire also asks about the child’s use of grammar and gestures.

An adaptation of the CDI was developed for use in the Mandinka language, following guidelines outlined by the MacArthur-Bates CDI Advisory Board. To construct an inventory of words for use in the Mandinka adaptation, a list of 200–250 words was compiled; these were taken from the standard CDI, the Malawian CDI and the Senegalese CDI. Mandinka-speaking field staff translated these words into Mandinka and suggested alternatives if words were not applicable in the West Kiang district ( *i.e.*, baby buggy/stroller) or more affected by seasonality ( *i.e.* some food items). The inventory probed specific categories from the MacArthur-Bates CDI, such as animals, food and drink, and clothing. It also asked whether the child had started to combine words and use more complex sentences. Subsequently, this inventory underwent pilot testing in two phases, with a total of 60 mothers of children aged 24 to 48 months. During the first phase, 30 women were interviewed, and the list was revised, removing words that were not frequently endorsed and adding new words that had been suggested by the pilot participants themselves. Subsequently, a second phase of pilot testing was conducted, where a further 30 women were interviewed. From these interviews, the list was reduced further by (1) eliminating all words which less than 10% of mothers said their child knew; (2) selecting 54 words of moderate difficulty (known by 40 to 70 % of the children); (3) selecting 18 easy words known by 70 – 100% of the children; and (4) selecting 18 more difficult words for which 10–40% of the children knew. This adaptation received full approval by the CDI committee as an official adaptation into the Mandinka language.

***Parental report sleep diary (adapted from the brief infant sleep questionnaire)***

A daily sleep log was administered over three consecutive days in the week prior to each lab visit and averaged over the monitored period at 1, 5, 8, 12, 18 and 24 months of age. This diary was adapted from the Brief Infant Sleep Questionnaire ( Sadeh, 2004) and methods for parental reporting ( Sadeh, 1996) into a three-day diary differentially for each site. In the UK the diary was sent to caregivers by post, and completed by caregivers, with an option to fill out an online version if preferred. The diary asked for a record of all periods of sleep (time and location) over a 72-hour period including information on anything that made the day/night unusual relative to their regular routine ( *i.e.*, illness/activity). In The Gambia the questionnaire was adapted in several ways following advice from the local ethics committees and through the formation of a consultation group comprised of local research staff who live and work in the West Kiang region of The Gambia and who had young children ( *i.e.*, to provide guidance on the range of locations parents might use for daytime naps). Firstly, the local population in West Kiang do not adhere to strict observance of equinoctial hours, nor do they necessarily possess a time piece in each home. Therefore, the diary was adapted so that caregivers could answer questions about sleep based around the sections of the day that are divided by prayer calls from the local mosques ( *i.e.*, morning, afternoon, evening, last prayer time). The prayer time calls differed slightly by season, which will be accounted for in analyses. Therefore, while fragmentation of sleep, location and number of daytime naps could be accurately recorded, length of sleep was restricted to an approximation. As with other questionnaires, the sleep diary was administered as an interview at the lab visit with caregivers asked to recall the previous three days and nights. While it would have been more accurate to interview caregivers day by day, the research team did not have the capacity for this many home visits. Families were, however, reminded to attend to their child’s sleeping patterns for three days prior to the study visit when they were notified about their visit date the week before the scheduled research visit.

***Questionnaires/interviews – Family***

**Family Care Indicators (FCI)**

In The Gambia, families were asked to complete the FCI questionnaire when the infants were 12, 18 and 24 months of age. The development of this set of indicators was initiated by UNICEF to provide measures of family care practises and resources with globally relevant application ( Kariger *et al*., 2012). The items measure the support provided by caregivers for a stimulating environment for infants to learn from, and the caregiving resources available within the home. For example, caregivers were asked about number of books and play items in the home, who was engaging with the child at home and how many different types of stimulating activities the child was encouraged to do. The questionnaire was developed by an international panel of experts who reviewed existing surveys used in low- and high-income countries ( *i.e.*, Home Observation for Measure of the Environment Inventory; Caldwell & Bradley, 1984) and field-tested new candidate questionnaire items across populations in five low-income countries before finalising this set of indicators for use in global health and epidemiological studies.

**Parental mental health**

Parental mental health was assessed using a range of questionnaires, starting at the antenatal visit and followed up until the 24-month time point. In the UK, the original English versions of the questionnaires were given to both parents (where applicable) to complete in their own time. In The Gambia, questionnaires were translated into Mandinka and administered in interview format (see below). Since mothers always accompanied infants to visits and fathers were often working away from home, we could only collect data on maternal mental health.

The Edinburgh Postnatal Depression Scale (EPDS) ( Cox *et al*., 1987) was administered at the antenatal, 1, 5, 12 and 24 month visits. This is a 10-item self-report questionnaire that asks participants to rate how frequently they have experienced a range of depressive symptoms in the last seven days. Items are scored on scale of 0–3 (“No, not at all” to “Yes, most of the time”) and possible scores range from 0 to 40. A cut-off of 10 is considered to indicate elevated levels of depression. The EPDS is a validated tool used to screen for postnatal depression and has previously been used to assess maternal mental health in The Gambia, as well as other LMICs ( Coleman *et al*., 2006; Nabwera *et al*., 2018).

The Positive and Negative Affect Schedule (PANAS) ( Watson *et al*., 1988) was administered at the 1, 5, 12 and 24 month visits. This 20-item self-report questionnaire asks participants to rate how frequently they have experienced a range of positive and negative emotions in the past few hours. Items are scored on a range of 1–5 (“Very slightly or not at all” to “Extremely”). There are six items that correspond to the Positive Affect (PA) and the Negative Affect (NA) scales, which are summed to compute scores for each scale, with a possible maximum of 30 for each scale.

The Percieved Stress Scale (PSS) ( Cohen *et al*., 1983) is a 10-item self-report questionnaire that asks participants to rate how often they have experienced a series of stress-related feelings in the last month. Items are scored on a scale of 0–4 (“Never” to “Very often”), with a possible total score of 40. The PSS has been shown to have robust psychometric properties across diverse low- and middle-income settings ( Katus *et al*., 2022a) and across different modes of assessment ( Murray *et al*., 2023). The PSS was administered at the antenatal, 1, 5, 12 and 24 month visits.

The Pregnancy Related Anxiety Scale (PRAS) ( Rini *et al*., 1999) and the Pregnancy Specific Anxiety scale ( Roesch *et al*., 2004) were used as measures of anxiety related to pregnancy at the antenatal visit only. The PRAS is a 10-item scale that asks respondents to rate how frequently they have experienced a range of concerns related to their pregnancy in the last few months. The scale is rated on a scale of 1–4 (“Not at all” to “Very Much” or “Never” to “A lot of the time”). The total score is computed by summing scores on all items, with a maximum score of 40 possible for the scale. The PSA asks participants to rate how often they have felt a range of emotions in the last week. Scores range from 1–5 (“Never” – “Always”). A total score is generated by summing the scores on four items that are specific to anxiety (anxious, concerned, afraid, panicky). Total scores can range from 4–20. The paternal versions of these questionnaires ask fathers to rate their feelings in reference to their partner’s pregnancy.

Over a period of eight months (2015–2016) each questionnaire was adapted following World Health Organization guidelines ( World Health Organization, 2013), the questionnaire developers, and procedures described in other studies using these measures in LMICs ( Hanlon *et al*., 2008; Kohrt *et al*., 2016; Nabwera *et al*., 2018; Tesfaye *et al*., 2010; Weobong *et al*., 2015). The adaptation protocol was the same for all measures and involved a core team of five researchers, as well as an additional nine staff members who supported this intermittently where needed. an initial translation from English to Mandinka by a panel of three Gambian research staff, the local PI (MD), who were all native speakers, and two researchers from the UK who were experienced in mental health data collection. Following best practice guidelines outlined by ( Peña, 2007), we attempted to align the translated items as closely as possible to the original English, while taking into account cultural equivalence. This involved replacing English idioms and medical terminology with phrases that would be comprehensible in Mandinka, and changing the structure of statements into questions ( Kohrt *et al*., 2016). The translation process involved several rounds of back translation by Mandinka-speaking staff who were blind to the original questionnaires and the translation process, as well as several meetings with local clinical staff ( *i.e.* midwives) and visits to families to discuss the language of the questions with mothers and pregnant women. Where discrepancies were noted between the original English and the back-translation, or regional variations in wording identified, the panel made necessary adjustments. The translated questionnaires were pilot tested with *N*=12 volunteers from West Kiang, to assess their understanding of the measures, corrections were made where issues with comprehension emerged.

While every attempt was made to ensure equivalence between the original English and the Mandinka translations, one item on the EPDS (item 10) that asked about suicidal ideation/behaviours was changed because of the highly sensitive nature of the question in this culture and local population. ( Nabwera *et al*., 2018) noted that, due to the highly communal way of life in this community, the desire to be isolated from others was seen as a sign that the individual may be suffering from a mental health problem. Therefore, this item was changed to ask participants whether they wanted to be isolated or alone. Participants who scored above clinical cut-off (a score of 10) on the EPDS were given the opportunity to be referred to the MRC clinic for support. Furthermore, some of the English words used, in the PANAS in particular, were not differentiable in Mandinka, and so the number of items were reduced in The Gambian version relative to the one administered in the UK.

To simplify administration and reduce recall of response options, mothers were first asked whether they had experienced the issue described in the question (yes/no) and, only if they responded with a yes, would the interviewer elaborate with the frequency options ( Hanlon *et al*., 2008). To help mothers remember the time period that each questionnaire was referring to, they were administered in order of timeframe, from shortest to longest, and the timeframe was reiterated with each question. Finally, to reduce the impacts of stigma, mothers were reminded that all participants were being asked the same questions.

Field staff responsible for administering the interviews received extensive training on understanding the conceptual framework of each measure. Subsequently, they were trained in administration using vignettes and role play scenarios, practicing administration and managing different types of potential responses.

***Socioeconomic status (SES), demographic and family information***

Families were asked to complete a questionnaire (UK) or interview (The Gambia) regarding their family demographics and socioeconomic circumstances. These were conducted as a series of questionnaires/interviews spanning from the first antenatal visit across the postnatal sessions, tailored to ask questions relevant to each time point, and with reduced time burden at each session, given that questions became an update on whether circumstances had changed. At both sites information was gathered on biological parents, biological grandparents (ethnicity, date, and place of birth) and any other applicable caregivers of the key participating infant/child. The caregiver information gathered included ethnicity, age, caregiving role, employment status, highest level of education, languages spoken. Wider family information was collected including parity of parents, size, and composition of household. Finally, housing information was collected pertaining to indication of wealth, *i.e.*, number of bedrooms (UK), wall/floor materials, access to water, durable assets (The Gambia). In The Gambia this information was gathered through a combination of observation by the field assistant during home visits and reports from the participant during interview.

***Pregnancy, birth and family health information***

In the UK and The Gambia mothers were asked to complete a questionnaire (UK) or interview (The Gambia) regarding their pregnancy, birth, and family medical history. Information gathered included: (i) antenatal information on maternal obstetric and medical history; (ii) fetal ultrasound information including gestational age and anthropometric foetal measurements (The Gambia only); (iii) birth and delivery information including neonatal anthropometric measurements (The Gambia only) and a maternal health check.

***Growth and diet measures***

**Anthropometric measures**

Anthropometric measurements were made by research assistants in the UK and by field assistants in The Gambia. In both sites, measurements were taken in triplicate, following standard protocols and all staff underwent training. In the Gambian sample, maternal height and weight were measured in late pregnancy and infant length, weight and head circumference were measured at birth. In addition, infant length, weight, head circumference, mid-upper-arm-circumference (MUAC) and knee-heel length were measured at both sites at 7–14 days, 1, 5, 8, 12 ,18 and 24 months of age. At birth, infant length was measured using a flexible length mat, and a fixed length board (SECA 417) was used thereafter. Infant weight was measured using a calibrated electronic baby scale (SECA 336), with a precision of 10g. Mid-upper-arm circumference and head circumference were both measured using a SECA 201 head and body measuring tape, precise to 1mm. Knee-heel length was measured using a calliper, also precise to 1mm.

**Dietary data**

In The Gambia, infant feeding data was collected every two weeks from birth to 24 months of age. The questionnaire was administered verbally by a field assistant at the participant’s home or by telephone if a home visit was not possible. The mother was asked to report on the infant’s diet in the two weeks prior to the questionnaire. Details included whether the infant received breastmilk feeds, and/or other liquids, semi-solid or solid foods. The questionnaire included common examples of local weaning foods, as well as free text space for additional items. The mother was also asked to report the frequency (never, once, more than once, most days) at which the infant received each food or drink.

In the UK, similar feeding questionnaires were completed monthly by parents online, from 1–7 months of age. In addition, parents completed a detailed food diary reporting all food and drink consumed by their infant, for four consecutive days prior to each study visit from 8 months onwards (8, 12, 18, 24 months). This data was then coded on a food composition database (DINO; ( Fitt *et al*., 2015)). Mothers also completed online 24-hour dietary recall questionnaires reporting on their own diets, in pregnancy and at 6 and 12 months postnatally, using the Intake24 UK platform.

**Biological samples**

In The Gambia, to investigate nutritional factors in more detail, samples of breast milk, blood and urine were collected. Maternal venous blood and urine were collected in late pregnancy (34–36 weeks’ gestation) and breast milk was collected from the mother at the five-month visit. Infant urine was collected at 5, 12 and 24 months of age. Samples were stored at -70°C for subsequent analysis. In addition, infant blood samples were collected at all infant visits, alternating between a 0.5mL capillary sample (at 1, 8 and18 months) and a 3mL venous sample (5, 12 and 24 months). On each sample, a full blood count was run using a Medonic analyser and the remaining sample was centrifuged. Plasma and cell pellets were separated and stored at -70°C for subsequent nutritional analysis and DNA extraction, respectively.

### Standardisation of protocol across sites

All measures were collected using Standardised Operating Procedures (available upon request). Longitudinal infant and toddler testing requires standardisation of the (i) equipment (ii) environment in which the measures are administered, (iii) experimental protocol, and (iv) behaviour of researchers during administration of measures. The site in The Gambia had not previously undertaken research of this kind until the pilot phase of the BRIGHT project ( Lloyd-Fox *et al*., 2014; Milosavljevic *et al*, 2019). Therefore, all equipment and testing materials had to be purchased prior to the start of the study sessions. To reduce site differences due to hardware, an identical set of equipment was purchased for both sites. While it is challenging outside of a research lab context to replicate the environment that the testing is undertaken in, where possible, we replicated the UK room setups at the Gambian site. In the UK (both at the hospital and university sites) testing rooms for visits from 1 – 24 months of age were sound proofed and windowless with temperature and lighting control. In The Gambia, the rooms were air conditioned to control the temperature, and the neurocognitive testing (fNIRS, EEG, eye-tracking) room was windowless with some light control. However, none of the rooms were sound-proofed. Therefore, environmental noise was more inconsistent across data collection within the Gambian sample as external sounds could sometimes be heard within the testing rooms. At both sites testing at 7 – 14 days of age was done at the family’s home, therefore environmental noise differed between the cohorts (for example family size was generally larger in The Gambia and houses often had open windows and doors– see Table 3). To address this, researchers at both sites optimised data quality where possible by discussing the needs of each measure with the family who were present during data collection ( *i.e.*, they discussed with the family that there would be times when they needed the room to be quiet during a measure of attention to sound or light, or when they might need their help in eliciting a smile from their baby)).

**Table 2.** Participant characteristics (age/sex) and retention rate of cohort at each time point. Note: If infant became tired or fussy before session was complete a call back was arranged for a second visit to complete testing; DOV = date of visit; N = sample size; SD = standard deviation of mean.

The Gambia (N = 214)							
Sex (female/male)	103/111						
Maternal age at birth Mean, SD (min-max)	29.76 (6.61), 18.2 – 44.7						
Timepoint	7–14 days	1 months	5 months	8 months	12 months	18 months	24 months
***Enrolled at DOV (N)***	205	204	200	193	192	191	185
Attended visit (N)	157	185	198	188	188	177	161
Mean age in days (SD)	12.3 (3.87)	36.0 (5.64)	159.9 (10.14)	247.2 (11.39)	372.7 (14.05)	558.6 (17.13)	745.0 (27.94)
Range in days	5 - 44	29 - 65	148 - 208	211 - 314	353 - 428	533-641	722-896
% attended visit	76.5	90.7	99.0	97.4	97.9	92.7	87.0
% attended two visits per age point*	n/a	3.2	22.2	23.9	25.9	37.9	16.8
UK (N = 62)							
Sex (female/male)	31/31						
Maternal age at birth Mean, SD (min-max)	32.96 (2.93), 28.4 – 40.8						
Timepoint	7–14 days	1 months	5 months	8 months	12 months	18 months	24 months
***Enrolled at DOV (N)***	62	61	60	60	60	57	50
Attended visit (N)	58	60	58	57	59	55	50
Mean age in days (SD)	12.2 (3.33)	33.2 (5.53)	155.8 (6.54)	251.7 (9.89)	375.7 (12.51)	557.1 (15.02)	736.9 (15.79)
Range (days)	7 – 23	22 – 56	144 – 184	235 – 279	353 – 411	536 – 603	700 - 784
% attended visit	93.6	98.4	96.7	95	98.3	96.5	100
% attended two visits per age point*	n/a	n/a	n/a	n/a	n/a	27.3	20

**Table 3.** Demographic characteristics and socioeconomic status, The Gambia. This socioeconomic information is derived from that reported or observed at the 7–14-day home visit. *n*=172; IQR = inter quartile ratio; NA = not applicable.

	Mothers (%)	Fathers (%)
**Education**		
No formal education	59.4	55.0
Some primary education	12.9	5.9
Complete primary education	3.5	4.7
Some secondary education	18.2	7.1
Complete Secondary education	5.9	27.2
**Household characteristics**		
Number of children, Median (IQR) min-max	5(4), 1-10	6 (7), 1-23
Number of wives	NA	1 (1), 0-4
**Assets**		
	Household (median (IQR), min-max)	
**Number of people in** **household**	11 (8), 3-36	
**Diet**		
Meals per week containing meat	1 (1), 0-5	
Meals per week containing fish	6 (1), 0-7	
Meals per week containing fish or meat	7 (0), 2-12	
Housing Attribute	Households (%)	
**Primary Water Source**		
Open Public Well	2.25	
Protected Public Well	7.87	
Public Tap	87.6	
Piped water in compound	2.25	
**Cooking Fuel**		
Firewood	96.1	
Charcoal	3.95	
**Toilet Facilities**		
Pit Latrine	96.07	
Improved Pit latrine	1.69	
Flush Toilet	2.25	
**Flooring**		
Earth, Sand, Mud	17.4	
Cement	70.2	
Vinyl	0.56	
Tiles	5.62	
Carpet	6.18	
**Roofing**		
Corrugate	100	
**Walls**		
Mud	12.9	
Earth Bricks	3.93	
Cement/ Burnt Bricks	82.0	
White Lime	1.12	
**Household Assets**		
Electricity	2.81	
Television	16.3	
Refrigerator	5.06	
Bicycle	73.0	
Motorbike	14.0	
Other vehicle	7.87	

Broadly, the protocol was identical across sites. As described above, some measures were site-specific either because a measure was found to be unsuccessful at one site ( *i.e.*, the tablet task) or because the measure is only relevant at one site ( *i.e.*, FCI). For the neurocognitive testing we adopted the use of the TaskEngine framework, which was developed for a separate multi-site neurocognitive study, Eurosibs ( Jones *et al*., 2019), to optimise data quality and standardisation of acquisition. This framework allows the presentation of the paradigms to be identical across sites and produces identical data outputs for cross-site quality control reports and analysis.

As described above, the order of testing was kept consistent across sites, however we have adopted the practice of standardization with flexibility to be responsive to the needs of the individual infant/toddler. Before the onset of the project, project coordinators for both sites were trained in the UK for a period of two months. Following this, training continued in The Gambia for one – two months (depending on the measure) and the first study sessions were conducted under supervision. The BRIGHT project team were committed to building long-term capacity for neurodevelopmental research at MRC Keneba and therefore across the duration of the project, trained and supported local researchers to conduct and co-ordinate all aspects of the research in The Gambia. To facilitate harmonization across sites, staff were trained in the practical detail of data collection and administration as well as researcher responsiveness to infant behaviour.

Working in partnership with local researchers during their training phase, we discussed the observed infant responses and behaviours elicited by each paradigm and used this experience to devise a standardized strategy for responding to the needs of the infant and caregiver. This strategy was agreed at both sites and included in the Standard Operating Procedures (SOPs). These steps took into account the infant’s state of alertness and fussiness, as well as the caregivers needs, to ensure optimal data collection and participant comfort. Significant fussiness typically leads to inadequate data quality across both the neurocognitive and behavioral measures. Fussiness was defined as excessive motion ( *i.e.*, infant wriggling on lap, or toddler walking away from task), inattention to the task ( *i.e.*, looking away) or negative affect ( *i.e.*, crying, negative vocalizations). Furthermore, particularly in The Gambia, a further category of inattentiveness existed as infants, on occasion, become drowsy or fell asleep during the task, likely an effect of the climate. We agreed upon a hierarchy of responses, tailored to the task, to ensure maximum participation. For example, during screen-based tasks ( *i.e.*, eye-tracking, fNIRS) to address possible boredom, when the infant began to fidget and look away, “attention-grabbers” (non-social short sounds) were employed to re-orient the participant to the screen. These were automatically recorded by the task presentation framework. If this was ineffective the researcher moved through the following strategies (also applicable to other measures); parents were asked to speak reassuringly and hold hands, the infant was given something to hold or at older age points infants/toddlers are offered a snack ( *i.e.* rice cake/rusk), a short break was offered before re-engaging with the task, or if none of these strategies were successful families were given a longer break for a nap or feed. If the parent was happy to resume the session continued. If a participant was unable to complete the full session within a day, families were asked if they were happy to return on a subsequent visit. All “manual” strategies of engagement and breaks for naps and food are recorded in the Session Log Form.

***Data analysis plan***

**Quality control**

Frequent refresher training sessions and quality control have been implemented across the duration of the BRIGHT project. At the Gambian site, due to the number of participants, time frame of testing and number of age points measured, testing load was very high at the peak of the project – with up to four infants tested per day, seven days per week, with staff on rotating schedules - for over two years. To maintain high data quality, research staff were trained to be highly specialised in a subset of measures rather than every measure. A researcher might oversee the neurocognitive tasks (EEG, fNIRS, eye-tracking), behavioural measures (MSEL, NBAS), mental health parent interviews or anthropometric measures. For example, for fNIRS studies, to minimize data loss, designated researchers were trained to monitor the system’s performance, detect potential problems with the acquisition and instructed to implement basic repairs ( Blasi *et al*., 2019). Designated researchers with expertise in a particular measure were also supervised by a senior team member with specialist knowledge of the measure or paradigm. The majority of the senior team members were located in the UK. From the start of the project, web-based multi-site meetings were routinely held. For the duration of the project, these consisted of fortnightly group meetings (which include senior team members and all designated researchers at the sites of data collection) and fortnightly quality control meetings (which included at minimum one senior team members and designated researchers). At these meetings recruitment, data quality, testing practices, outputs and other issues were discussed. Furthermore, during the quality control meetings training sessions were also conducted, including researcher responsiveness, as and when the need arose. For example, during the life course of the project, training in the MSEL had to be administered as each new age point was reached to ensure the data was being collected in a standardised way across sites. During the BRIGHT-Kids phase of the project, training on all measures was complete, therefore, team meetings were reduced to fortnightly quality control meetings until completion of data collection.

After data collection, each data set required extensive quality control assessments.

For example, EEG and NIRS data needed to be assessed for artefact from motion and infant inattentiveness, and segments of data removed as appropriate. Session level data from the “Session Log Form” was also reviewed so that contextual variables such as infant state ( *e.g.*, fussiness), interference from researcher/caregiver ( *e.g.* talking during task), experimenter error ( *e.g.* video not recorded), technical issues ( *e.g.* computer program crashed, headgear misaligned for NIRS/EEG), or position of participant ( *e.g.* infant facing wrong way, or parent out of view during interaction video) could be taken into account. Data was marked for validity at each stage of this process to monitor data attrition during the stages of the processing stream. Data quality metrics were extracted for all paradigms at each site and age point. Specific guidance for conducting EEG and fNIRS studies in global health contexts to maintain high quality control measures can be found in two methods papers concerning the BRIGHT project ( Blasi *et al*., 2019; Katus *et al*., 2019)

***Data storage and handling***

While it would be preferable for data pre-processing and analysis to be conducted at the site of data acquisition, such skills are highly specialised and usually attributed to post graduate education in neuroimaging and such expertise among local researchers in The Gambia is, as of 2023, very limited. This is likely because developmental neuroscience is a very new field of research in The Gambia, and as such, opportunities to learn the required analytic skills have previously been lacking. This has been identified as an important focus for capacity building in the long term. However, to ensure timely data quality control during the BRIGHT project, much of the data analysis was conducted within the UK and a reliable data transfer method had to be established to enable this. A dual protocol was designed to ensure the integrity of the data transfer.

As a standard procedure, initially, the data was stored and backed-up locally on site in a separate location to the research data acquisition computers. Personal data ( *i.e.*, contact details, DOB) was stored securely at each site within locked cabinets as well as within a password encrypted electronic database isolated from the research data. Data from the anthropometric measures, parent-report questionnaires, and behavioural assessments ( *i.e.*, NBAS, MSEL) was pseudonymised and housed in password-protected encrypted databases locally. All data obtained using paper forms was double entered on the local databases to ensure reliability across staff. Experimental data ( *i.e.*, eye-tracking, neuroimaging measures, parent-infant videos) was pseudonymised and stored on password encrypted storage hard drives at each local site. Following this, a Secured File Transfer Protocol (SFTP) server functioned as a bridge between sites. This system allowed the transfer of data in both directions to account for (i) planning of protocol updates, (ii) software and stimuli transfer from the development site (London, UK) to cohort sites (Cambridge, UK and Keneba, The Gambia), (iii) data transfer from the cohort site and (iv) feedback on pilot data and quality control checks (see Blasi *et al*., 2019 for further detail). A second full copy of all research data was transferred to a secure password encrypted server in one of the participating UK centres (at the Centre for Brain and Cognitive Development, Birkbeck, University of London).

One consistent challenge was the unreliable internet and/or poor bandwidth, which lead to slow transfer times for large files. The fNIRS tasks of the BRIGHT project, for example, involved acquiring over 160 files, equating to over 3GB of data, in total across all time points for each participant ( Blasi *et al*., 2019). Furthermore, during the life course of the project over 25,000 data files were collected for the infant/child measures alone (this excluded datasets for anthropometric data, biological data and family questionnaire data). Therefore, when the rate of data transfer became restricted, we prioritized the transfer of specific data types based on the data quality control checks and analysis pipelines required for each.

Data transfers, quality control checks and inventories were carried out at regular intervals. Access was fully audited, and to ensure data security, access is governed by a management team. A web-based interface enables internal BRIGHT researchers to access the database using personalized login details to search, filter, and download data. Data access is overseen by the data management team in the UK and The Gambia, and access is granted for internally pre-registered projects (see Data.

***Data analysis and statistical plan.***

Overall, we aim to identify developmental brain and neurocognitive function-for-age curves across the first two years of life, establish which context associated moderators ( *i.e.*, under nutrition, family income, caregiving/family support) impact significantly on infant development and how these associate with pre-school outcomes at three to five years. Further to the plans outlined for each aim below, tests of normality and sensitivity analyses (comparing observed values and imputed missing values) will be conducted. Non-linear tests of significance and interpolation approaches will be applied where appropriate.

To address Aim 1, brain and neurocognitive function-for-age curves from birth to 24 months of age will be generated across 1, 5, 8, 12, 18 and 24 months of age. These will be derived from the fNIRS, EEG, MSEL and neurocognitive eye-tracking batteries of tasks. These reference curves will be used to enable age-adjusted group comparisons of differences in average trajectories, group-wise differences in variability, and for characterizing the range of individual developmental trajectories within each cohort. To generate appropriate metrics for each dataset we will explore which type of derivative measures and the level of complexity required to meaningfully capture brain and cognitive change across this developmental window. For the neuroimaging data we will explore these derivative measures ( *i.e.*, localisation *versus* globalisation of brain response, latency of response, profiles of change in haemoglobin) by running time varying parameter models to explore variation across and within these values over our varying age points. Following this, several approaches will be undertaken, including longitudinal growth modelling to explore relationships between measures, and regularisation methods such as latent function connectivity modelling, to explore whether we can derive a common measure across the different brain function tasks which predict rate of change in other measures such as MSEL and the neurocognitive battery.

To address Aim 2, a combination of quantitative analyses will be undertaken to establish the association between context-associated moderators ( *i.e.*, undernutrition in mother and infant and consequent growth faltering, parental mental health, SES factors) and developmental trajectories across the first two years of life in The Gambia. To generate appropriate metrics for each dataset we will explore which type of derivative measures most meaningfully capture measures of poverty ( *i.e.* maternal iron status *versus* infant iron levels, physical growth at birth, *versus* change in growth measures over time, SES measures of income, household size, household assets). Structural equation modelling (SEM) and hypothesis-driven regressions will explore how these latent pre- and post-natal variables associate with latent outcomes of infant development and regression analyses conducted to understand the directional relationships between outcome variables.

To address Aim 3, outputs from Aims 1 and 2 will be nominated using lasso regression coefficients in relation to longitudinal brain and cognitive development trajectories ( *i.e.* across language, motor, sensory, attentional correlates, brain connectivity,) across 0–24 months of life using SEM, applying full information maximum likelihood to account for missingness and to identify developmental-hypothesis driven clusters to explore how context-associated moderators (including risk factors of poverty) of altered infant development, and the impact of these in turn on pre-school outcome measures of early child development.

## Preliminary results

### Recruitment and retention of participants: The Gambia

**Figure 3.**
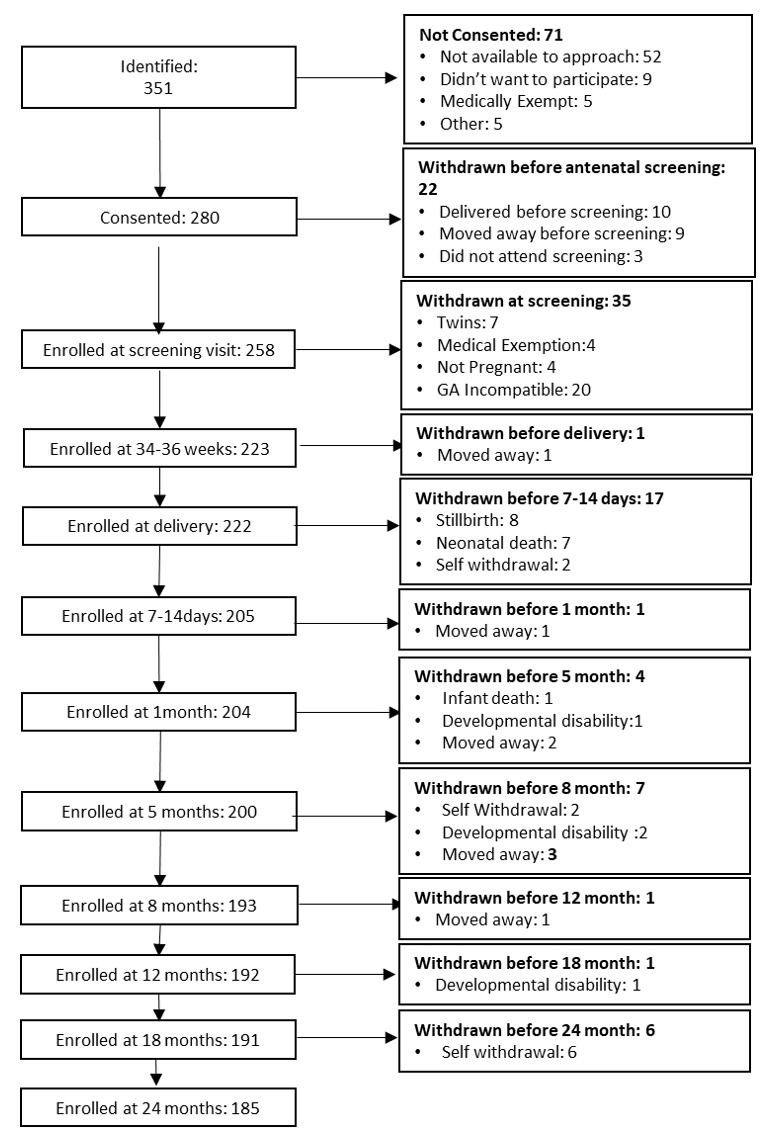
Recruitment and retention of participants, The Gambia.

Figure 3 illustrates the recruitment and retention of participants in The Gambian cohort. In total 280 families were recruited and consented into this project. A total of 58 families were excluded from the study prior to delivery, as outlined in Figure 3, leaving a total of 222 families enrolled at delivery. Eight infants were stillborn, leaving a total postnatal cohort of 214 mother-infant dyads. A further seven infants were lost to neonatal death during the first two weeks of life and two families chose to withdraw from the study prior to the first postnatal home visit at 7–14 days of infant age, leaving 205 mother infant dyads within the study. At the 24-month time point, 185 families remained enrolled within the study.

### Recruitment and retention of participants, UK

**Figure 4.**
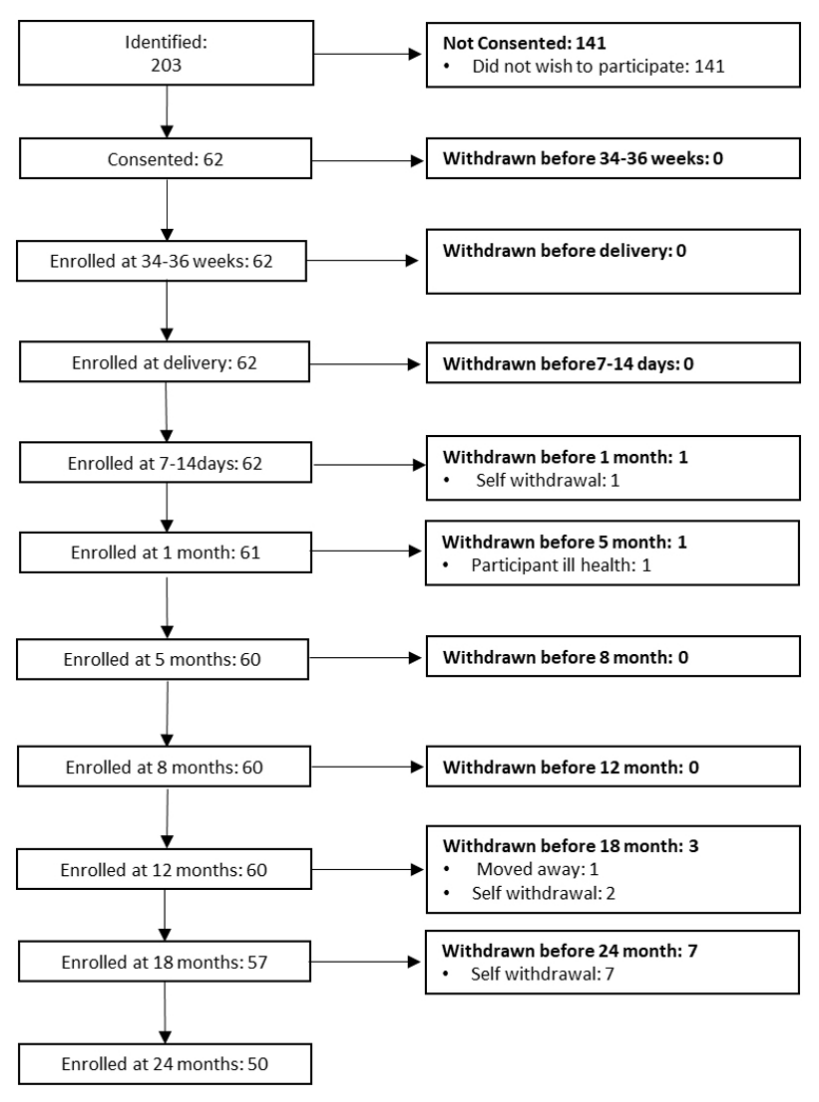
Recruitment and retention of participants, UK.

Figure 4 illustrates the initial recruitment process in the UK, and the retention of participants throughout the study. In total, 62 families were recruited and consented into this study, undertook the antenatal assessment and took part in their first postnatal home visit when the infants were 7–14 days of age. Participating families live either in the centre of Cambridge (N = 22) or in surrounding urban or rural communities within a 20-mile radius (N = 40).

### Participant attendance to study visits

This data was derived from the first data collection point and includes all available data for N = 214 (The Gambia) and N = 62 (UK). At each age point a preferred testing window was established; +/- 1 week at 7–14 days, +/- 2 weeks at 1 – 8 months, +/- 1 month at 12 – 24 months. To minimise data loss, we allowed data collection up to + 1 month at 5 – 8 months, and + 2 months from 12 months upwards. This became necessary on occasion, particularly if both parents returned to work or when families had travelled outside of the region and were unavailable at the time of test. The total number of participants who attended each visit is given in Table 2. Due to the COVID-19 pandemic, testing of the full 24-month time point was suspended in The Gambia during their first country-wide lockdown. Following a prolonged period of suspension of research, a decision was made to end data collection for the BRIGHT project, therefore 20 participants could not be invited for their final time point at 24 months of age. For those families enrolled at each time point ( *i.e.*, excluding those who had been withdrawn for health reasons or because they moved away) we have experienced a high retention rate for the majority of the completed time points (> 90% of enrolled cohort attended visits). In The Gambia the exception has been at the 7 – 14 days visit, where the proportion of families attending the visit fell to 76.6%. This occurred as a result of mothers who gave birth away from their village and would therefore stay with other family members during the first weeks of their infant’s life. Consequently, it was sometimes challenging to identify that these women had given birth and arrange their first visits. Furthermore, during late 2016 – early 2017 the country experienced political unrest during the general elections, which impacted on our ability to schedule infants at 7–14 days and 1 month of age for their visit. In the UK, retention rates have been high in the first time points, for example 91.9% of participants were seen at all five of their visits during their first year of life. While the majority of those families were still enrolled in the study at 18 and 24 months of age and attended the visit, we experienced higher rates of self-withdrawal in the UK as the testing burden on some participating families became too high (the most common reasons for withdrawal were either that both parents had returned to work or that the mother was expecting/had given birth to a further child).

### Demographic characteristics and socioeconomic status (SES), The Gambia.

A summary of the family demographic and SES distribution of The Gambian cohort is given in Table 3. Families live in multigenerational households with up to 36 members per compound. Polygamy was common within the cohort with 38.8% of fathers having more than one wife. Consequently, while mothers had on average 4.4 children, including the infant enrolled in the study, fathers had on average 6.9, with a range of 1 – 23 children attributed to a single father.

For the generation of parents within our cohort, formal schooling was readily available when they themselves were children, therefore, on average, mothers and fathers within the study had completed three and four years of schooling respectively. Over five times more fathers (26.6%) than mothers (5.62%) had completed high school (grade 12). Subsistence farming was the most common profession among both mothers (64.4%) and fathers (43.4%). In terms of livestock, fathers owned more livestock (sheep, goats, donkeys, or cows) than mothers. Goats were the most commonly owned livestock among both mothers and fathers, with 45.5% mothers and 46.6% fathers owning at least one goat. Cows are the most valuable livestock, and ownership was heavily skewed in favour of the fathers with 29.7% of fathers compared to 6.7% mothers owning at least one cow. Around one third (34%) of farming fathers sold a proportion of their produce, however the mothers’ farm produce was largely consumed by the household, with only a small minority (6%) of mothers selling any of their harvest. Durable asset ownership was low, while 73.3% of households reported owning a bicycle, only 14% owned a motorbike, 7.9% owned a vehicle, 16.3% a television and 5.1% a refrigerator.

**Figure 5.**
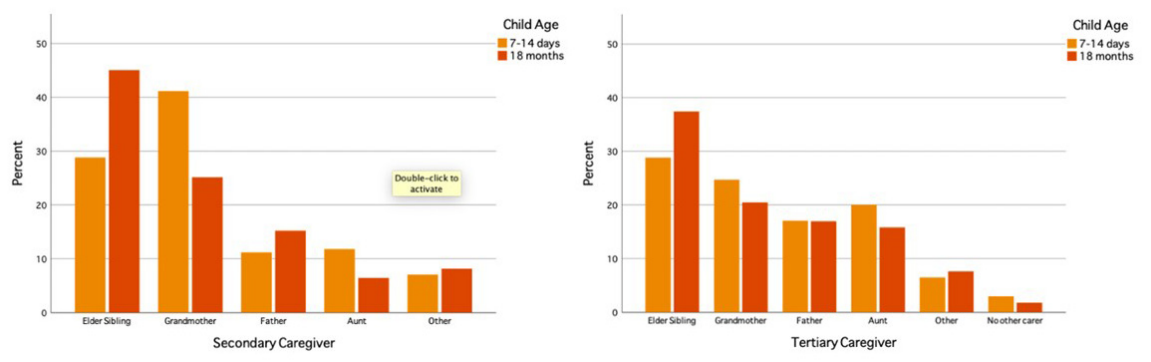
Additional caregivers at 7–14 days and 18 months of age, The Gambia. The Figures display the make-up of secondary and tertiary caregivers at 7–14 days and 18 months of age in The Gambian cohort. Secondary caregiver; a person who looks after the infant a substantial proportion of the time. Tertiary caregiver; a person who looks after the infant ‘sometimes’. A. n=170, B. n=165, C. n=171, D. n=168.

Within the cohort, the mother was reported to be the primary caregiver for all infants at 7–14 days of age, and for all but one child, where the primary caregiver was reported as the aunt, at 18 months of age. As can be seen in Figure 5, at 7–14 days of age, the most common secondary caregiver was the grandmother (41.1%), whereas by 18 months almost half (45.0%) of all participants reported an elder sibling to be the secondary caregiver. Fathers were reported as secondary or tertiary caregivers in 28.2% of families at 7–14 days, and in 32.2% of families when infants were 18 months old.

**Figure 6.**
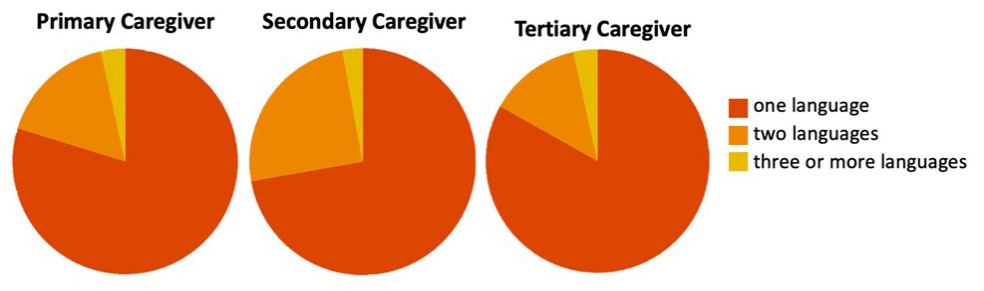
Number of languages spoken by caregivers, The Gambia. The Figures show the proportion of primary (n=172), secondary (n=171) and tertiary (n=167) caregivers at 18 months of age, who spoke one, two or three languages.

All primary caregivers reported that their first language was Mandinka. In addition, 16.7% of primary caregivers reported that they spoke a second language and 3.5% of primary caregivers spoke three languages. Mandinka was also the most common first language among secondary and tertiary caregivers, the only exceptions being three participants for whom their tertiary (n=2) or secondary and tertiary (n=1) caregivers’ first language was an alternative local language. In addition, 25% of secondary caregivers and 13% of tertiary caregivers spoke a second language and a small minority spoke a third language (2.3% and 4.1% of secondary and tertiary caregivers respectively) ( Figure 6). Other than Mandinka, languages spoken included local languages such as Fula, Wolof and Jola, as well as English and Arabic.

### Demographic characteristics and socioeconomic status (SES): UK

A summary of the family demographic and SES distribution of the UK cohort is given in Table 4.

**Table 4.** Demographic characteristics and socioeconomic status, UK. Note that this summary is compiled from the 18 month visit; SD = standard deviation of the mean.

	Mother (%)	Father (%)
**Education**		
Secondary	3.2	6.5
Tertiary	3.2	4.8
Undergraduate	33.9	33.9
Postgraduate	54.8	48.4
**Family Ethnicity**		
Caucasian	87.1	83.9
Asian	3.2	6.5
Black	1.6	1.6
Mixed/Other	3.2	3.2
**Parental SOC Classification**		
Higher managerial or professional	69.8	76.8
Intermediate	22.6	19.6
Routine/semi-routine	3.8	3.6
Unemployed > 6 months	3.8	0
	Household (mean (SD), min-max)	
**No of children (incl. key child)**	1.19 (0.4), 0-3)	
Annual Household Income	Percent of Households (%)	
£20,000 - £29,999	1.6	
£30,000 - £39,999	1.6	
£40,000 - £59,999	25.8	
£60,000 - £79,999	37.1	
£80,000 - £99,999	21.0	
£100,000 - £149,999	6.5	
> £149,999	1.6	
Do not wish to answer	3.2	

**Figure 7.**
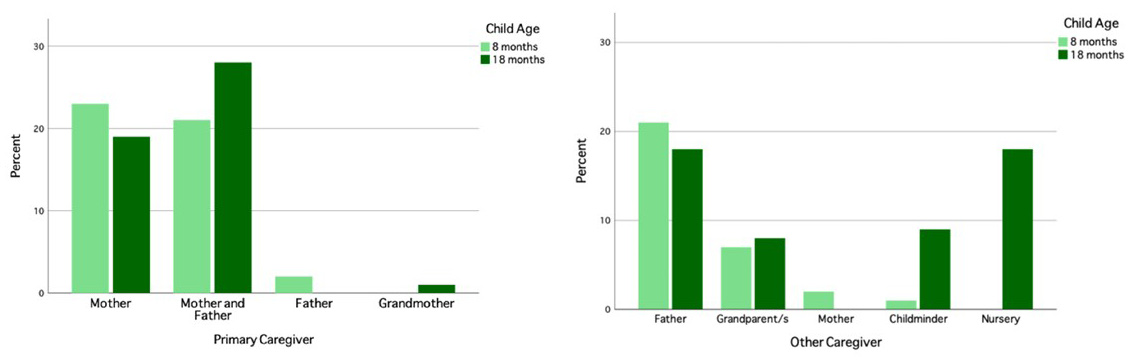
Caregivers reported at 8 and 18 months of age, UK. The Figure shows the primary caregivers and other reported caregivers at 8 and 18 months of age for the UK cohort. 8 months of age n=46, 18 months of age n=48.

**Figure 8.**
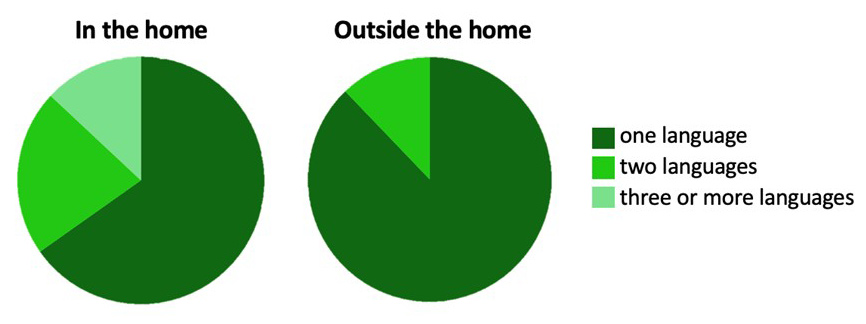
Number of languages heard in the home by infants at 18 months.

As can be seen in Figure 7, at 8 months of age, caregiving in the UK cohort was primarily provided by the mother and/or father. Other reported caregivers at this age included grandparents and a childminder. Similarly, at 18 months of age, caregiving was primarily provided by the mother or jointly by the mother and father, with the exception of one family who reported the grandmother as the primary caregiver. Nursery workers, childminders and grandparents were also frequently reported as additional caregivers at this age ( Figure 8).

**Figure 9.**
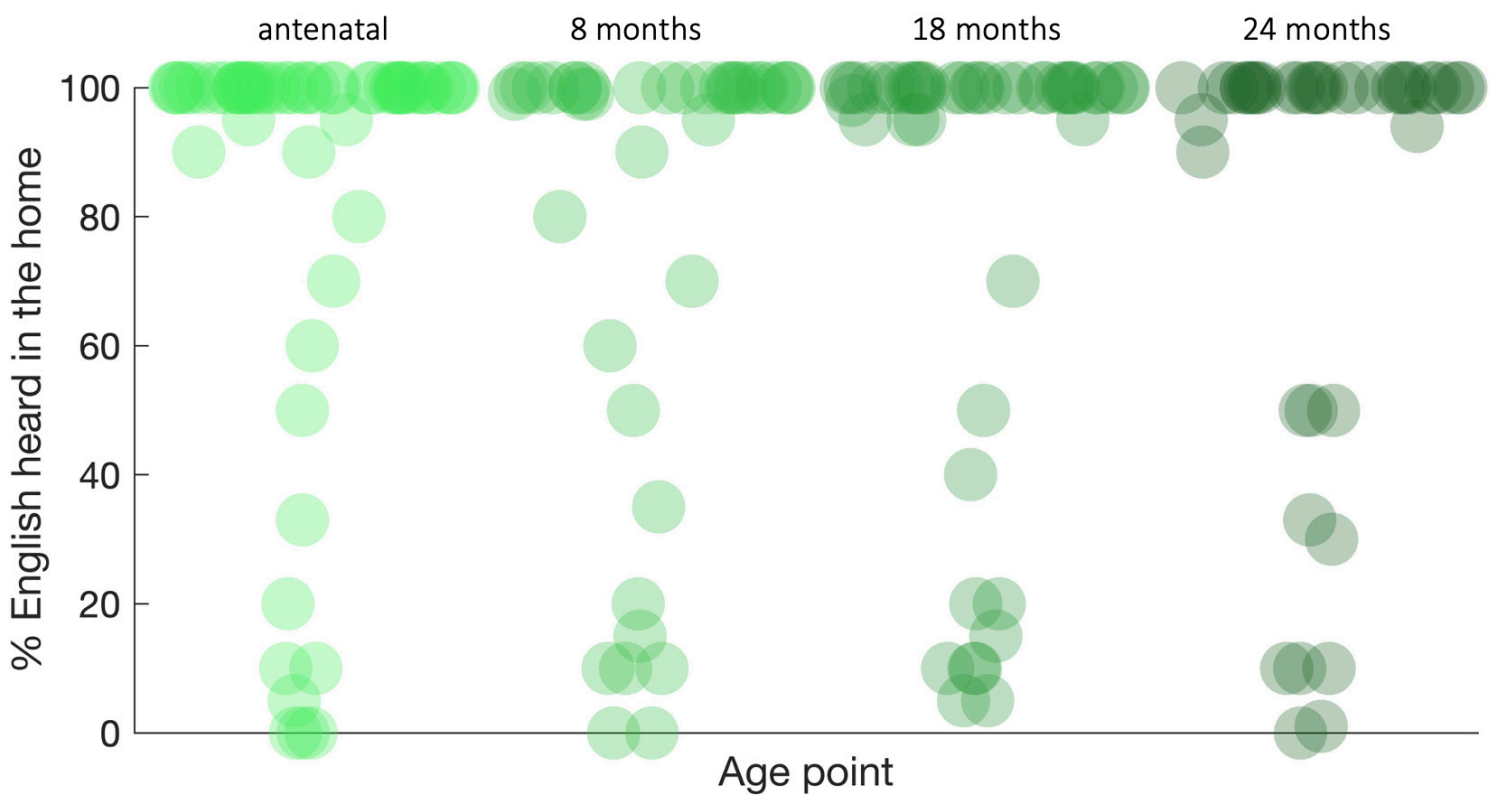
Percent of English heard by UK infants across age points. Note: While the majority of families completed this questionnaire antenatally (95.2%), the proportion of families who provided this information at 8 – 24 months dropped significantly to 62.9 – 74.2% complete datasets from the full cohort. This was due either to families withdrawing from the study at 18 – 24 months or missing data within the demographic questionnaire.

In the UK we followed a recruitment strategy that encompassed natural population variance within the region of recruitment. A large proportion of the population living in the city of Cambridge and surrounding areas is multi-lingual. Consequently, a significant proportion of our recruited infants were exposed to multiple languages ( Figure 8). As highlighted in Figure 9, exposure to languages both within, and outside of, the home was recorded antenatally and then at 8, 18 and 24 months of age. Within the home, the proportion of households exposed to English-only varied from 55 – 72.9% across age points. A further 13.6 – 27.5% of households had one additional language, 10 – 15.4% had two additional languages and 0 – 7.5% of infants were not exposed to English within the home at all. The languages that the infant was exposed to outside of the home differed with 75.6 – 85.7% of families reporting that infants were exposed only to the English language outside of home across 8 – 24 months of age. A further 11.4 – 14.7% reported that their infants heard English and one additional language outside of the home, one further family reported that their infants heard two other languages in addition to English, and one family reported that their infant did not hear English outside of their home at all. At this stage we wish to avoid premature consideration of cultural and linguistic factors that could affect the interpretation of our results within the UK, given that this was not a primary focus of our project. However, this factor will be taken into account during our planned analyses.

## BRIGHT Kids: Implementation, challenges, and successes

The protocol for BRIGHT Kids is outlined below and summarised in Table 5.

**Table 5.** Protocol for BRIGHT-Kids. Summary of abbreviations; fNIRS, functional near infrared spectroscopy; EEG/ERP, electroencephalography/event related potentials; L – length, W – weight, H – height, HC – head circumference, MUAC – mid to upper arm circumference, KHL – knee to heel length. * Indicates a new measure introduced for BRIGHT-Kids.

Measure	
Neuroimaging measures	Questionnaires/Interviews – Infant/Child
fNIRS: Social/Non-social	Food Insecurity Q (FFQ) *
fNIRS: Habituation and Novelty Detection	Dietary Diversity Q (IFQ) *
fNIRS: Functional connectivity	Early Childhood Development Index *
EEG: Auditory Oddball	Questionnaires/Interviews – Family
Behavioural/Neurocognitive measures	Generalised Anxiety Disorder – Maternal *
Eye-tracking: Cognitive control	Patient Health Questionnaire – Maternal *
Eye-tracking: Gap/Overlap	Perceived Stress Scale (PSS) – Maternal
Eye-tracking: Non-social contingency	Impact of COVID-19 *
Eye-tracking: Face popout	Family Care Indicators (FCI)
Eye-tracking: Dynamic scenes	Clinical measures/ Medical details
Eye-tracking: Word-picture-matching	Anthropometric measures – infant (L,W,HC,MUAC, KHL)
Mullen Scales of Early Learning (MSEL)	
Tablet-based Early Years toolbox *	
Test of Gross Motor Development *	

### Aims of BRIGHT Kids

While a growing body of research has begun to identify early markers of risk and resilience, the consequences of adversity often do not become fully manifest until later in childhood. Preschool age is a period where children experience rapid progress in a number of developmental domains ( *e.g.*, language, executive functions), as well as an increase in external demands from caregivers. Consequently, this is a period where delays in development start to become outwardly observable and to interfere with everyday functioning. Thus, to fully understand the consequences of early exposure to context-associated moderators, it was necessary to continue tracking the development of children in The Gambian cohort beyond infancy and into the preschool period.

The preschool age follow-up (hereafter “BRIGHT Kids”) has several key aims: (i) to assess a broader, age-appropriate set of developmental outcomes; (ii) to further elucidate developmental trajectories of the measures that had been administered since infancy; and (iii) to examine associations between biomarkers of risk in infancy and outcomes at preschool age.

To examine these aims at the BRIGHT Kids follow-up, we implemented a combination of existing and new measures, described below. Prior to inviting the BRIGHT sample for the follow-up, we recruited a separate pilot cohort of 24 participants to evaluate the implementation of the new measures into the protocol.

### Participant retention, sample size and age distribution

In 2021, all participants that had attended the 24-month visit were invited to take part in the BRIGHT Kids follow-up. Of these, 181 provided informed consent for participation. Two families were traveling and could not be reached and two declined to participate. A further three families withdrew from BRIGHT Kids after consenting and six remained in the study but did not attend the scheduled visits. Finally, one child was withdrawn from the study due to evidence of developmental delay (which interfered with their ability to complete any of the assessments). This left a final sample of 171 (49.7% female) participants that took part in BRIGHT Kids.

**Figure 10.**
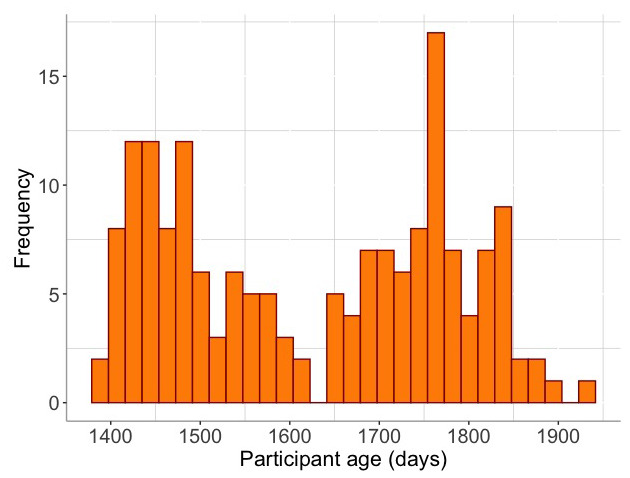
Distribution of participant age (in days) in the BRIGHT Kids follow up.

As a result of over recruitment in the early stages of the BRIGHT project and interruptions to testing caused by a political crisis in December 2016–January 2017 (which resulted in the short-term closure of MRC Keneba), a recruitment pause was needed in the summer of 2017. This recruitment pause gave the number of infants born into the study per month a bimodal distribution. Furthermore, the initial BRIGHT-Kids phase of the BRIGHT project was conducted over a compressed time window relative to the earlier longitudinal time points of the BRIGHT project, due to funding and pandemic related restrictions during 2021–2022. Children were assessed starting with the oldest (first recruited) and ending with the youngest (last recruited). Therefore, the ages of children at follow-up reflected the same bimodal distribution as the number of infants born per month. The age range of participants in Phase I of BRIGHT-Kids was 45–63 months ( *M*=52.8, *SD*=5.06); see Figure 10.

With the support of additional funding, due to this bimodal distribution of ages, a Phase II data collection of BRIGHT-Kids, has now been established to collect data during 2023. This additional follow-up is designed to assess the younger half of the age distribution again when they are age matched with the older participants from Phase I. The same BRIGHT-Kids protocol is being utilised to allow for us to assess potential outcome and exposure effects by collecting data from all participants when they reach 4 – 5 years.

### Protocol for neuroimaging, ET, and MSEL

A number of paradigms that had previously been used in the BRIGHT protocol were retained in BRIGHT Kids. These included fNIRS tasks (social cognition, habituation and novelty detection, functional connectivity), EEG, ET tasks and the MSEL. The aforementioned assessments were either developed using stimuli that was suitable for broader age ranges throughout early childhood (fNIRS, EEG, ET) or had specific items suitable for preschool-aged children (MSEL). Using consistent paradigms enabled further tracking of developmental trajectories, which is informative because it tells us whether a particular biomarker is relevant at only one developmental/age period, or if it continually predicts outcomes throughout early childhood ( Loth *et al*., 2017). While the neuroimaging paradigms were suitable for the age group tested, several practical considerations were required to ensure suitability of the headgear.

**fNIRS headgear**

Data acquired at this visit were collected using a headgear based on an EasyCap, rather than the custom-built headband that were used at prior visits. These caps are made of soft fabric that covers the whole head. The optodes were held in place with purpose-made holders for a secure fit on the head and the design was changed to have a pointy, rather than flat, end to better penetrate hair in older children. Both of these changes allowed for better data acquisition, particularly considering the variety of hair types at this age.

**Practical considerations**

One challenge of undertaking fNIRS and EEG with older children is that they have more hair than infants, and children (girls in particular) often have their hair tied or in braids ( Katus *et al*., 2019). Several steps were taken to maintain data quality and account for a variety of hair types. If participants had large braids, mothers were asked to undo these before the study visit. When fitting the fNIRS or EEG caps, research staff brushed aside stray hair to ensure that the optodes/electrodes made contact with the scalp. To acknowledge this additional burden on participating families, and to thank them for their time, we hired an assistant, who was local in West Kiang, to act as a “hairdresser” for the participants. At the end of the assessments, she washed out the EEG gel from the children’s hair and re-braided the hair of every child (with caregiver consent).

***Protocol for additional behavioural measures***

**Gross motor skills**

The *Test of Gross Motor Development 3 ^rd^ Edition* TGMD-3 ( Ulrich, 2019) was used to assess gross motor (GM) skills. The MSEL does not provide age-appropriate test items, nor does it describe developmental GM milestones, for children older than 39 months ( Mullen, 1995). The TGMD-3 is suitable for children aged 3–11 years and assesses GM skills across two domains: locomotor and ball skills. The locomotor subscale tests skills that require coordinated and fluid body movements ( *e.g.*, running, skipping, hopping). The ball skills subscale evaluates proficiency in throwing, catching and striking movements. Age-normed scores can be computed for both scales, which are then combined to form the Gross Motor composite score.

**Tablet-based executive functioning assessments**

Tablet based assessments from the Early Years Toolbox ( Howard & Melhuish, 2017) were used to assess executive functions (EFs) across three domains: working memory (“Mr Ant” task), Inhibitory Control (the Go/No-Go task) and Cognitive Flexibility (Card Sorting task). These tasks were previously used among preschool aged children (3–5 years) in South Africa, who demonstrated good EF abilities and, on some scales, outperformed their Australian counterparts ( Howard *et al*., 2020). Task instructions were presented as an audio playing from the tablet. These had been translated into Mandinka and recorded by field staff and embedded into the app. While the tablet task was challenging at the earlier time point, we found this tablet task to be successful in The Gambia with our cohort at 3 – 5 years ( Milosavljevic *et al*., 2023) .

**Parent-report measures**

We continued to use the Family Care Indicators (FCI, described above) to assess play materials, enrichment activities and caregiving. In addition to this, a number of new questionnaires were implemented to assess child development, caregiver wellbeing and the home environment.

**Child development**

The *Early Childhood Development Index (ECDI2030)* is a caregiver report questionnaire that assesses the achievement of fundamental developmental milestones among children aged 24–59 months. The measure was developed by UNICEF with the aim of creating a tool that could be both nationally representative and also provide internationally comparable data on child development (for full details, see the UNICEF ECDI resources). The questionnaire consists of 20 items that assess a range of skills related to a learning, health, and psychosocial wellbeing. Since a Mandinka version was not available, we translated the tool following the customisation and translation guidelines outlined by UNICEF.

**Maternal mental health and caregiving**

Maternal wellbeing has important implications for child wellbeing and development beyond infancy and into school age ( Bennett *et al*., 2016; Kingston & Tough, 2014). Therefore, we continued to assess maternal mental health at the BRIGHT Kids visits. The Perceived Stress Scale (PSS, described above) continued to be used, as this assessment is not specific to pregnancy and was, thus, deemed suitable for this time point. In addition to this, two new assessments of depression and anxiety that were designed to assess these mental health constructs more generally and were not specific to pregnancy, were introduced. The Generalised Anxiety Disorder-7 ( Spitzer *et al*., 2006) and the Patient Health Questionnaire-9 (PHQ-9; Kroenke *et al*., 2001) were implemented to assess anxiety and depression, respectively. These measures were translated and adapted following the same method as the previously used mental health assessments. For the adaptation of these two measures, we collaborated with the PRECISE-DYAD study, who were assessing maternal health in a different area of The Gambia (for further details see PRECISE-DYAD website) to create a Mandinka version of these measures that could be used in multiple sites and studies. As with the previous measures, these were administered as interviews. The two measures are described in more detail below.

The GAD-7 is a seven-item self-report questionnaire that asks respondents to report how often they experienced a range of anxiety-related symptoms in the last two weeks. Responses are rated on a Likert scale ranging from 0–3 (not at all-nearly every day). The scale generates a total score, which can range from 0–21. There is also one item assessing the severity of symptoms, where respondents are asked to report how much the symptoms that they reported add difficulty to their daily lives on a scale of 0–3 (not at all to very difficult). The GAD-7 has previously been used in Kenya ( Nyongesa *et al*., 2020), where it was reported to have good psychometric properties and the same factor structure as the original English. For our translations, all of the items were deemed to be appropriate for the local setting by the field staff working on the translations.

The PHQ-9 is a nine-item self-report questionnaire that asks respondents to rate how often they experienced a range of depression-related symptoms in the last two weeks. Responses are rated on a Likert scale ranging from 0–3 (not at all-nearly every day). Scores can range from 0–27 and different ranges indicate varying severity of depression (0–4 – none; 5–9 – mild; 10–14 – moderate; 15–19 – moderately severe; and 20–27 – severe). Similar to the GAD-7, the PHQ-9 has one item assessing severity of symptoms, where respondents are asked to rate how much difficulty they experience in their daily lives as a result of reported symptoms on a scale of 0–3 (not at all to very difficult). Mothers who report elevated levels of depression were offered an opportunity for referral to the on-site clinic, as was done at previous visits. The field team found most of the items on this scale suitable for the local population. However, as with the EPDS, there were concerns about asking participants to report on suicidal thoughts. Thus, as for the EPDS, this item was changed to asking participants how often they wanted to be alone or isolated.

**Impact of COVID-19**

The BRIGHT Kids follow up was conducted during the COVID-19 pandemic and, thus, it was vital to better understand how the pandemic impacted on each family’s wellbeing, financial situation and access to healthcare. The questionnaire was adapted from a measure that was being used in a UK study at the time to assess the impact of the pandemic on families with young children ( Aydin *et al*., 2022). Items that were deemed by field staff to be irrelevant or unsuitable for the West Kiang community were removed. The final measure consisted of a series of questions that asked the caregiver to report on how their living and financial situation had changed due to the pandemic, whether they had family members who got sick with COVID-19, their ability to access healthcare, and their general feelings and concerns related to the situation.

**Nutrition and child growth**

Measures of child physical size, namely height, weight, and head circumference, continued to be measured at this visit (see above for details). In addition to this, measures of family food insecurity and the child’s dietary diversity were also implemented.

The *Dietary Diversity Questionnaire* ( Swindale & Bilinsky, 2006)) is a caregiver report questionnaire that asks respondents to indicate what the child eats on a typical day, from 12 food groups. There is also an option for “miscellaneous”. The *Food Insecurity* questionnaire asks two sets of questions. The first comprises of a single item that asks respondents if anybody in the family has missed a meal due to food shortage in the last seven days. Subsequently, there are seven items asking respondents to indicate any food shortages in the household over the previous month.

## Discussion

### Good scientific practice

We recognise the importance of maximising outputs from the data collected in the BRIGHT project, both by serving the participants and communities that have agreed to partake in this research and the wider scientific community by providing access to the collected data for further analysis. Access to any data collected during or generated by the BRIGHT project is fully audited, and to ensure data security, is overseen by the data management team in the UK and The Gambia. While data sharing is critically important to maximising the benefit of research, we must also consider the need to protect the confidentiality of this sensitive group (particularly the infants within the mother-infant dyads, who as minors do not consent for themselves). Furthermore, to generate maximum value from this dataset we must link data points together ( *i.e.* NIRS/EEG data with outcome data or contextual factor data). Due to the nature of the data being collected ( *i.e.* collected from a specific geographical location, longitudinal dataset of several datapoints) the majority of the data cannot be fully de-identified under the guidance included in the European General Data Protection Regulation (GDPR). Furthermore, some BRIGHT measures include photo, audio and video material, and therefore is inherently identifiable and requires even stricter governance. In line with other EU based studies of this nature ( *i.e.* EUROSIBS, ( Jones *et al*., 2019)), currently, access to the data sets for the wider scientific community is governed by the data management team to ensure that users comply with all relevant data protection laws and have appropriate ethical permissions. Access operates via a project approval form, which is reviewed by a committee consisting of representatives from each cohort site, and implements relevant data sharing agreements. Collaborations are encouraged, and projects are evaluated primarily on their consistency with the ethical principles and aims of the project that the families signed up to when partaking in this study. All planned analyses (both internal to the BRIGHT team and external) are pre-specified either on an internal database monitored by our management committee or via web-based pre-registration platforms. These procedures continue to be evaluated annually and updated to optimise the BRIGHT Project’s value to the scientific community and public priorities.

Dissemination of key findings has, and will continue, to take place, through presentation of findings by the BRIGHT research team at national and international conferences, internal reports and peer reviewed publications hosted on our website, and through direct interaction with key stakeholders and public engagement events.

### Lessons learned

During the life course of the BRIGHT Project (and the pilot phases that preceded this) we have encountered many challenges and opportunities that provide us with important guidance for future work. Firstly, the importance for harmonisation of goals, sense of purpose and motivation across the research team and study sites should not be underestimated. Only through our dedicated research team, which includes the 46 authors of this paper, and countless more research midwives, clinical staff, lab assistants, village community assistants, drivers, mechanics, (even a hairdresser for the children’s hair) and research centre staff have we managed to succeed in achieving what we have thus far in the BRIGHT Project. In particular in The Gambia, staff coordination was critical while we managed the study workload across the time course of the study with the research team working on rolling interleaved working hours so that the participants could be tested seven days a week during the peak period of longitudinal timepoints. Furthermore, a strength of our study came in the establishment of rigorous SOPs – adapted for each site, age point and measure obtained – and overseen by the quality control and data handling procedures and regular meetings outlined above. The pilot phase of our project enabled us to identify areas where sites differed and discuss with the research team at each site how to adapt best to the contextual factors that were relevant. An important area was to operationalise our management of infant behaviour to ensure successful collection of neuroimaging and behavioural infant measures. We broadly harmonised these approaches and used a series of sequential “attention getters” within task, and calming strategies (such as a snack, break or comforting social strategies) to maintain successful data collection, with levels of attrition being broadly similar across sites and measures. However, there were some differences noted across sites. In The Gambia for example, the climate required us to more carefully monitor environmental conditions, a significant rise in temperature could lead the infant to become drowsy and fall asleep during the session at a more frequent rate at some age points relative to the UK. In the UK, in contrast, we found that age related changes in the behaviour of individuals led to some losing attention more frequently and moving around or leaving their parent’s lap at older ages relative to younger. While in The Gambia this age-related behavioural shift was less evident. It is possible that some of these site differences exist due to fluctuations in the testing practice of the research team, or differences in caregiving approaches or infant motivational behaviours that led to differences in performance during the sessions, or differences in other contextual factors of the sample at each site (for example the proportion of infants and families having a stressful or long journey to the research site). It is hard to tease apart these differences systematically, but our approach to data analysis will include provision for these contextual factors. It should also be noted that heterogeneity can be a strength, and individual variance within sites as well as across sites can be used to further our understanding of infant and early childhood development. As noted by the EUROSIBS consortium, “multisite studies need to consider balancing the resources put into standardisation with the desirability of testing whether metrics are robust across natural variation between sites” ( Jones *et al*., 2019).

Building on this, a strength of our project comes in our approach to incorporating contextual information in the design, development and adaptation of measures to different contexts. We were faced with a need to adapt measures to be broadly applicable across different cultural and linguistic contexts, while also ensuring we designed site specific measures when appropriate. While many of these measures had been used extensively in the UK, and other HICs, not all of these had been used across all the age points of the BRIGHT protocol, so piloting work focused on ensuring that the questionnaires and paradigms were relevant across the developmental time frame that we are studying at both sites. Furthermore, in the Gambia, our approach was to ensure Gambian research staff co-produced the necessary adaptation of measures and assessed the appropriateness of tools in a context that they had not been used previously to take advantage of the local expertise at each site. Furthermore, staff in The Gambia are also involved in data processing and quality control to ensure that, when possible, our training strategies incorporate not just data collection knowledge transfer but also steps towards transferring knowledge on data analysis and dissemination. The latter is certainly an area for future development, as this area of research (developmental psychology/neuroscience) is relatively new to the country and so capacity building is a key priority for our group. While we strive to be sensitive to cultural context, a limitation of some of the measures used in this project is that they are drawn from a pool of existing developmental measures of cognitive outcomes which have largely been derived from tasks developed for high-income settings. While we went to lengths to adapt these measures, we cannot fully eliminate possibility of cultural bias. Equally, these measures likely overlook some skills that are relevant in a rural, farming community as many measures have been designed in urban contexts, therefore we cannot be certain that we are measuring all of the crucial predictors of SES ( Hermida *et al*., 2019). For example, some studies have defined preschool attendance and access to services as a better predictor of cognitive outcomes than income. In focussing on poverty initially, we also overlook many of the strengths of this community and culture, which are highly relevant. While we strive to understand potential protective factors in this environment, we may not have fully captured the complex family dynamics that exist, and will focus on furthering our understanding in future work.

A final strength of the BRIGHT project comes from the diversity of measures incorporated in the cohort design and the density of time points. The spacing of study visits and the range of cognitive, brain and contextual measures employed means that we are able to more accurately pinpoint when certain skills emerge and when they start to become relevant for later outcomes. Likewise, we are also able to investigate whether a particular marker is relevant throughout childhood or only during a specific age/time in development.

### Future efforts

The ultimate objective of the next phase of the BRIGHT project is the identification and validation of a marker, or “fingerprint” combination of brain function markers, that predict the contribution of exposure phenotypes ( *i.e.*, undernutrition / caregiving context) to the substantive variation in developmental outcomes seen in infants born into a low-income setting, such as rural Gambia. Innovative modelling and analytic approaches will be applied to allow us to address the following aims: (1) To employ feature extraction and time varying parameter modelling to identify the most reliable “neural fingerprinting” biomarkers (from fNIRS and EEG) of longitudinal developmental cortical specialization; (2) To create a profile of metrics across developmental outcomes in later childhood (executive function, language, general cognitive development, adaptive skills) at the group level and identify latent classes that sub-group individuals into developmental profiles of pre-academic skills; (3) To expand our predictive models of longitudinal fNIRS and EEG trajectories of early developing brain networks (0–2 years) to determine which age points and “fingerprint” of biomarkers are the most consistent and reliable predictors of developmental outcomes of language, pre-academic skills and functional brain specialization in later childhood (3–5 years); (4) To model exposure phenotypes and establish health, social and environmental risk and resilience determinants of developmental trajectories from birth to preschool which associate with early biomarkers of development, to identify primary targets of intervention.

## Conclusions

The BRIGHT project is a comprehensive, and multi-method study of development, during the first two years of life in the UK and the first five years in The Gambia. The combination of neuroimaging, behavioural, parent-report measures, and biological samples provides a unique opportunity to study a variety of context associated moderators, which may, or may not be related to poverty, as well as the mechanistic processes underlying associations between markers and outcomes. On one hand, we hope that our work will be an asset to global health research, where the study of neurocognitive development in early infancy, particularly with the use of neuroimaging tools, is still emergent. On the other hand, this work has broader value for developmental research in general. Due to the logistical and financial constraints of longitudinal research, our project is among the few to assess early development, across a large number of study visits, and that incorporates such a variety of methods. Thus, the generated results will enable us to identify critical windows for developmental vulnerability and act as rationale to guide future interventions which aim to protect and enrich the developing brain within context associated risk contexts. We propose that our project provides a roadmap for other researchers interested in conducting studies of neurocognitive development in LMICs with similar contextual factors.

## Figures and Tables

**Table:** 

Contributor Role	Role Definition	Authors
Conceptualization	Ideas; formulation or evolution of overarching research goals and aims.	Main Authors: SLF, BM, SEM, CEE BRIGHT Study Team: TA, MDH, AP
Data Curation	Management activities to annotate (produce metadata), scrub data and maintain research data (including software code, where it is necessary for interpreting the data itself) for initial use and later reuse.	Main Authors: BM, SMC, LK, AB, MCL, GG, LM, MP, CB, EM, ET BRIGHT Study Team:
Formal Analysis	Application of statistical, mathematical, computational, or other formal techniques to analyze or synthesize study data.	Main Authors: SLF, BM, SMC, LK, AB, MCL, GG, LM, CB, MS, ON BRIGHT Study Team:
Funding Acquisition	Acquisition of the financial support for the project leading to this publication.	Main Authors: SLF, BM, LK, SEM, CEE BRIGHT Study Team: TA, MDH, AP
Investigation	Conducting a research and investigation process, specifically performing the experiments, or data/evidence collection.	Main Authors: SMC, BM, LK, SLF, AB, MCL, GG, TF, EM, FN, ON, MP, MR, FS, MS, ET BRIGHT Study Team: LA, ABa, CBT, SB, DC, MC, YD, NH, ED, SDa, SD, AJ, SJ, BJ, MJ, OK, KK, JL, LS, LSt
Methodology	Development or design of methodology; creation of models.	Main Authors: SLF, BM, LK, AB, MCL, LM, SEM, MR BRIGHT Study Team: CBT, MDH, JL
Project Administration	Management and coordination responsibility for the research activity planning and execution.	Main Authors: SLF, BM, SMC, MCL, EM, MP, MR, ET, SEM, CEE BRIGHT Study Team: LA, TA, SB, NH, SD, PN
Resources	Provision of study materials, reagents, materials, patients, laboratory samples, animals, instrumentation, computing resources, or other analysis tools.	Main Authors: SLF, CEE, SM, BRIGHT Study Team: TA, EC, AF
Software	Programming, software development; designing computer programs; implementation of the computer code and supporting algorithms; testing of existing code components.	Main Authors: AB, LM, CB, BM, SMC, LK BRIGHT Study Team:
Supervision	Oversight and leadership responsibility for the research activity planning and execution, including mentorship external to the core team.	Main Authors: SLF, BM, SMC, LK, AB, CB, MCL, EM, LM, MR, SEM, CEE BRIGHT Study Team: LA, CBT, SB, MDH, SD
Validation	Verification, whether as a part of the activity or separate, of the overall replication/reproducibility of results/experiments and other research outputs.	Main Authors: SLF, AB, BM, MCL, CB, SCB, BRIGHT Study Team:
Visualization	Preparation, creation and/or presentation of the published work, specifically visualization/data presentation.	Main Authors: SLF, BM, SMC, LK BRIGHT Study Team:
Writing – Original Draft Preparation	Creation and/or presentation of the published work, specifically writing the initial draft (including substantive translation).	Main Authors: SLF, BM, SMC, LK BRIGHT Study Team:
Writing – Review and Editing	Preparation, creation and/or presentation of the published work by those from the original research group, specifically critical review, commentary or revision – including pre- or post-publication stages.	Main Authors: SLF, BM, SMC, LK, AB, CB, MCL, GG, TJ, EM, LM, FN, ON, MP, MR, FS, MS, ET, SEM, CEE BRIGHT Study Team: LA, TA, ABarjo, CBT, SB, DC, MC, EC, YD, MDH, AF, NH, ED, SD, AJ, SJ, BJ, MJ, OK, KK, AP, PN, LS, LStein

## Data Availability

No data are associated with this article. We recognise the importance of maximising outputs from the data collected in the BRIGHT project, both by serving the participants and communities that have agreed to partake in this research and the wider scientific community by providing access to the collected data for further analysis. Access to any data collected during or generated by the BRIGHT project is fully audited, and to ensure data security, is overseen by the data management team in the UK and The Gambia. While data sharing is critically important to maximising the benefit of research, we must also consider the need to protect the confidentiality of this sensitive group (particularly the infants within the mother-infant dyads, who as minors do not consent for themselves). Furthermore, to generate maximum value from this dataset we must link data points together ( *i.e.* NIRS/EEG data with outcome data or contextual factor data). Due to the nature of the data being collected ( *i.e.* collected from a specific geographical location, longitudinal dataset of several datapoints) the majority of the data cannot be fully de-identified under the guidance included in the European General Data Protection Regulation (GDPR). The data used to support this study are stored in the Brain Imaging for Global Health Data Repository. The conditions of our ethics approval do not allow public archiving of pseudonymised study data. The data cannot be fully anonymized due to the nature of combined sources of information, such as neuroimaging, sociodemographic, geographic and health measures, making it possible to attribute data to specific individuals, and hence, falling under personal information, the release of which would not be compliant with GDPR guidelines unless additional participant consent forms are completed. Our data sharing procedures were created in consultation with stakeholders and external consultation ( Begum-Ali *et al.*, 2023). Collaborations are encouraged, and projects are evaluated primarily on their consistency with the ethical principles and aims of the project that the families signed up to when partaking in this study. All planned analyses (both internal to the BRIGHT team and external) are pre-specified either on an internal database monitored by our management committee or via web-based pre-registration platforms. These procedures continue to be evaluated annually and updated to optimise the BRIGHT Project’s value to the scientific community and public priorities. To access the data, interested readers should contact the BRIGHT coordinator on the Contact page of our website. Access will be granted to named individuals following ethical procedures governing the reuse of sensitive data. Specifically, requestors must pre-register their proposal, and clearly explain the purpose of the analysis so as to ensure that the purpose and nature of the research is consistent with that to which participating families originally consented. Additionally, requestors must complete and sign a data sharing agreement to ensure data is stored securely. Approved projects would need to adhere to the BRIGHT project’s policies on Ethics, Data Sharing, Authorship and Publication. Legal copyright restrictions do not permit us to publicly archive the full set of behavioural tests and task paradigms used in this experiment. Readers seeking access to these tests are advised to contact the lead author or the reference list. No part of the study procedures or analysis plans was preregistered prior to the research being conducted.
