# Supplementary material for: The Brain Imaging for Global Health (BRIGHT) Project: Longitudinal cohort study protocol
Source: Gates Open Res. 2024 Sep 5;7:126. Originally published 2023 Oct 18. [Version 2] doi: 10.12688/gatesopenres.14795.2 (PMC11452580; doi:10.12688/gatesopenres.14795.2)
Supplement: Peer review history

# Reviewer report: Reviewer response for version 2

**DOI:** 10.21956/gatesopenres.17325.r39043

**Published:** 2025-01-09

**Authors:** Linda Larson-Prior

**Affiliations:** 1 University of Arkansas for Medical Science, Little Rock, USA

In this study protocol, the authors describe the development and implementation of a cross-cultural, longitudinal study of infant development from birth to 24 months of age in the UK and The Gambia.  Additionally, a study expanding the study population to include pre-school aged children is presented together with both new and previously utilized test protocols.  The authors have fully responded to previous review, and now include more detail on testing platforms, study questionnaire implementation and publications from study phase 1.  The study design is comprehensive and based on clearly articulated study objectives.  The inclusion of multi-modal neuroimaging assessments will provide quantitative data on neurodevelopment that could not be collected using surveys, questionnaires, anthropomorphic or demographic information and is a major strength of the study design.

 The authors carefully detail study protocols and data collection methods, clearly describing changes in study protocols that were needed when applying tools that were developed for use in high income countries or were culturally inappropriate.  The presentation of methods for porting research instruments to the local language, and for harmonizing protocols between the two study sites (UK and The Gambia) are also clearly presented.

 While the authors clearly describe the reasons why they are unable to publish their data in public access repositories and indicate that partial access can be provided to qualified research teams, how they plan to share data and even what data they will be able to share is not described.  For example, the website hyperlinked under “Discussion: Good scientific practice” provides no information on either current publications related to the BRIGHT and BRIGHTKids studies or on contact points for those wishing to either collaborate on or utilize data from these studies.  As is appears that there is no webpage specifically pointed toward a data repository or data access point, the authors are encouraged to provide information on whether and how data sharing will be accomplished.  Further, the Data Availability section references a data repository that I was unable to find on internet search.

Is the study design appropriate for the research question?

Yes

Is the rationale for, and objectives of, the study clearly described?

Yes

Are sufficient details of the methods provided to allow replication by others?

Yes

Are the datasets clearly presented in a useable and accessible format?

Partly

Reviewer Expertise:

I am a neuroimaging and neurocognitive research scientist who directs the Budding Brain Laboratory at the Arkansas Children's Research Institute where I study the impact of diet and physical activity on cognitive development from birth to adolescence.  At the University of Arkansas for Medical Sciences, my laboratory focuses on cognitive function in older adults, with a focus on Parkinson's disease

I confirm that I have read this submission and believe that I have an appropriate level of expertise to confirm that it is of an acceptable scientific standard.

# Reviewer report: Reviewer response for version 2

**DOI:** 10.21956/gatesopenres.17325.r37930

**Published:** 2024-10-04

**Authors:** Prerna Aneja

**Affiliations:** 1 University of East Anglia, Norwich, England, UK

I have gone through the amendments and they look good to me. I don't have any additional comments on the paper

Is the study design appropriate for the research question?

Yes

Is the rationale for, and objectives of, the study clearly described?

Yes

Are sufficient details of the methods provided to allow replication by others?

Partly

Are the datasets clearly presented in a useable and accessible format?

Yes

Reviewer Expertise:

NA

I confirm that I have read this submission and believe that I have an appropriate level of expertise to confirm that it is of an acceptable scientific standard.

# Reviewer report: Reviewer response for version 1

**DOI:** 10.21956/gatesopenres.16121.r35766

**Published:** 2024-02-27

**Authors:** Rihui Li

**Affiliations:** 1 University of Macau, Taipa, Macao

This study protocol introduces the detailed design and implementation of the Brain Imaging for Global Health (BRIGHT) project, which primarily aims to study the longitudinal change of brain function, cognitive development, and growth within the rural Gambian population. The protocol also aims to find out the critical developmental moderators, mediators, and markers of risk and resilience by comparing the Gambian cohort with the UK cohort. I think this is an exciting project that makes use of a variety of brain imaging techniques and behavioral assessments to collect multi-dimensional data. I have several minor suggestions to be considered.

 It may be beneficial to list the specifications of the EEG, fNIRS system as well as the eye-tracking device used in this project, such as channel number, sampling rate, and whether short channels are used. Such information could help to justify the reliability and feasibility of the protocol.

 A few neuroimaging tasks (e.g., working memory, habituation) are used in this project, please provide more information about these tasks.

 Psychiatric disorders, such as Autism or anxiety disorder, may be potential factors affecting brain and cognitive development. I wonder whether the research team is capable of screening and excluding those participants.

 Since the Phase 1 of BRIGHT is completed 3-4 years ago (ran from 2015-2020), I recommend the authors add a section or/and table to briefly describe relevant publications and main findings in order to highlight the value of the BRIGHT project.

Is the study design appropriate for the research question?

Yes

Is the rationale for, and objectives of, the study clearly described?

Yes

Are sufficient details of the methods provided to allow replication by others?

Yes

Are the datasets clearly presented in a useable and accessible format?

Yes

Reviewer Expertise:

neuroimaging, neuropsychiatry, social interaction, biomedical engineering

I confirm that I have read this submission and believe that I have an appropriate level of expertise to confirm that it is of an acceptable scientific standard.

# Response

**Published:** 2024-05-12

**Authors:** Sarah Lloyd-Fox

We thank you for your helpful and constructive comments. We have addressed all comments in our revision and believe this has strengthened the manuscript. Below we provide detailed responses to the individual points raised (your points in bold, ours in standard or italicised. 

**This study protocol introduces the detailed design and implementation of the Brain Imaging for Global Health (BRIGHT) project, which primarily aims to study the longitudinal change of brain function, cognitive development, and growth within the rural Gambian population. The protocol also aims to find out the critical developmental moderators, mediators, and markers of risk and resilience by comparing the Gambian cohort with the UK cohort. I think this is an exciting project that makes use of a variety of brain imaging techniques and behavioral assessments to collect multi-dimensional data. I have several minor suggestions to be considered.**

1. **It may be beneficial to list the specifications of the EEG, fNIRS system as well as the eye-tracking device used in this project, such as channel number, sampling rate, and whether short channels are used. Such information could help to justify the reliability and feasibility of the protocol.**

**Response: **We agree that these details would help contextualise our protocol and findings further. As outlined below, we have added additional detail to the relevant sections:

 fNIRS:

*This multi-domain battery was designed to interrogate whether global health risk factors impact on development to result in global/cross-domain differences in brain activity or localized/domain-specific differences or altered function. Data were collected using the NTS optical imaging system (Gowerlabs Ltd. London, Everdell *et al.*, 2005), which emits near-infrared light at the 780nm and 850nm wavelengths with a sampling rate of 10Hz. The total array consisted of 18 channels at 1 month, 34 channels at 5, 8, 12, 18 and 24 months. The same 34-channel layout was used in BRIGHT kids.  Channel separation was kept constant at 20mm across age points. Co-registration information from age and head size matched MRIs was used to adjust the placement guidelines of the headgear to ensure the channels were aligned over the frontal and temporal regions at each age point.*

 EEG:

*Due to the nature of the sounds (pure tones, bursts of white noise etc.), no adaptations had to be undertaken allowing for identical protocols at both project sites. Infants were presented with auditory stimuli for approximately 15 minutes while asleep (at 1 month) or awake (at 5 and 18 months): during the latter an experimenter quietly entertained the infant with bubbles or silent toys to maintain calm attention during the task. We used a low-density (8 electrodes) gel based Neurolectrics Enobio EEG system with passive electrodes with a sampling rate of 500Hz.*

 Eye tracking:

*A series of pilot studies were then completed in both the UK and The Gambia to identify population- and age- appropriate word-picture stimulus pairs. Data were collected using a Tobii TX-300 (Tobii AB, Sweden) remote eye tracker, set to a sampling rate of 60Hz.*

 2. **A few neuroimaging tasks (e.g., working memory, habituation) are used in this project, please provide more information about these tasks.**

**Response: **We agree that such detail would be helpful to others wishing to implement similar task protocols. To not overload the manuscript with detailed methodological descriptions for all components of this extensive protocol, we have opted to more clearly signpost prior work (both our own and other groups’) to provide easily accessible links to the specific tasks used.

- *EEG:* *(for a description of the EEG task and full protocol see* *Katus et al., 2020)*
- *fNIRS: The fNIRS paradigms implemented in the BRIGHT project assessed a range of cognitive functions and domains, namely social cognition* *(see Lloyd-Fox et al., 2017 for details of task)* *, habituation and novelty detection* *(see Lloyd‐Fox et al., 2019)* *, working memory (using an adapted version of the task described in Begus et al., (2016)), deferred imitation (see Katus et al., (2022) for task description) and functional connectivity (see Bulgarelli et al., (2024)).*
- *Eye-tracking:* *The tasks used assessed cognitive control (Wass et al., 2011), habituation (Katus et al., 2019), attentional disengagement using the Gap/Overlap task (Holmboe et al., 2010), non-social contingency (Delligianni, 2011), preference for faces (Gliga et al., 2009), and spontaneous looking (de Urabain et al., 2017).*

3 . **Psychiatric disorders, such as Autism or anxiety disorder, may be potential factors affecting brain and cognitive development. I wonder whether the research team is capable of screening and excluding those participants.**

**Response: **We agree that the conditions highlighted by the reviewer might affect the responses measured in this project. Infants who had suspected developmental delay or disability were referred for medical review and, as outlined in our exclusion criteria, those diagnosed with a developmental disability were excluded from the study. However, because autism and anxiety are typically not diagnosed until later in childhood, and due to the lack of appropriate medical expertise and follow up care, we did not screen for these conditions. The following is included in the main text:

*Infants with suspected developmental delay or disability were referred for medical review and, as per the exclusion criteria, infants/ children who were diagnosed with developmental disabilities (e.g. Down’s Syndrome and cerebral palsy) were excluded from the study. Previously collected data relating to these participants was removed from the dataset. Specific screening for further psychiatric disorders including autism and anxiety disorder was not completed as (i) these conditions are usually diagnosed later in childhood and (ii) appropriate necessary medical expertise and follow up care was not available. Further screening will be taken into consideration if the cohort is followed up in the future.*

 4. **Since the Phase 1 of BRIGHT is completed 3-4 years ago (ran from 2015-2020), I recommend the authors add a section or/and table to briefly describe relevant publications and main findings in order to highlight the value of the BRIGHT project.**

**Response: **We added this information in the Table below, to provide a more accessible summary of key findings obtained to date:

*We have multiple publications that describe both the methods development and pilot testing that was conducted prior to the start of the project, as well as publications from the data collected in BRIGHT (please see Table 6 for list and summary of BRIGHT publications to date).*

 Table 6 overview for your benefit in this review reply (is formatted properly in the resubmission file).

 Info given in the following order:

**Citation**

**Title**

**Data examined**

**Main findings**

 Lloyd-Fox et al., 2019

 Habituation and novelty detection fNIRS brain responses in 5- and 8-month-old infants: The Gambia and UK.

 fNIRS habituation and novelty detection at 5 and 8 months in The Gambia and the UK

 Differential profiles of habituation and novelty detection across the two cohorts.

 Katus et al., 2019

 Implementing neuroimaging and eye tracking methods to assess neurocognitive development of young infants in low- and middle-income countries

 NA

 fNIRS, EEG and eye tracking show robustness for use in a less well-controlled lab setting in The Gambia. We address common challenges related to implementing a multi-method protocol longitudinally.

 Blasi et al., 2019

 fNIRS for Tracking Brain Development in the Context of Global Health Projects

 NA

 Review of the use of fNIRS in the context of global health studies. We discuss the implementation of fNIRS studies in LMICs with a particular emphasis on the Brain Imaging for Global Health (BRIGHT) project, and we consider its potential in this emerging field.

 Katus et al., 2020

 ERP markers predict neurodevelopmental outcomes in young infants in rural Africa and the UK.

 EEG auditory oddball data at 1 and 5 months, MSEL data at 5 months in UK and Gambia

 UK and Gambian cohort both show immature novelty response at 1 month of age. A novelty response emerges at group level in UK, but not in the Gambian cohort. The emergence of a novelty response is associated with concurrently MSEL scores at 5 months of age.

 Collins-Jones et al., 2021

 Longitudinal infant fNIRS channel-space analyses are robust to variability parameters at the group-level: An image reconstruction investigation.

 fNIRS Social paradigm at 5, 8 and 12 months in The Gambia

 Traditionally, fNIRS analysis is done in the channel space, where data from equivalent channels across participants is combined, assuming that head size and source and detector positions are constant across participants. We conclude that channel -space analysis of longitudinal fNIRS data is robust to assumptions about head size and array position given the variability in these parameters in our dataset.

 Katus et al., 2022

 Neural Marker of Habituation at 5 Months of Age Associated with Deferred Imitation Performance at 12 Months: A Longitudinal Study in the UK and The Gambia.

 EEG auditory oddball data at 1 and 5 months, deferred imitation data at 8 and 12 months of age in UK and Gambia

 EEG habituation indices at 5 months of age were associated with imitation responses at 12 months of age in both cohorts. In the Gambian cohort, EEG novelty responses at 5 months were also associated with imitation responses as 12 months of age.

 Katus et al., 2023

 Longitudinal fNIRS and EEG metrics of habituation and novelty detection are correlated in 1-18-month-old infants.

 EEG auditory oddball data and fNIRS habituation and novelty detection data at 1, 5 and 18 months in Gambian cohort only.

 fNIRS and EEG responses were correlated for habituation at 1 and 5 months of age and for novelty detection at  5 and 18 months of age.

 Milosavljevic et al., 2023

**Executive functioning skills and their environmental predictors among pre-school aged children in South Africa and The Gambia**.

 Executive Functioning (EF; working memory, inhibitory control, and cognitive flexibility) collected at 3-5 years in the Gambia, and a South African sample of the same age. Performance on these tasks was examined in relation to task norms, and family enrichment factors (enrichment activities, diversity of caregivers) and socioeconomic status (SES), and caregiver educational attainment.

 The Gambian and South African participants showed normative performance across EF tasks, with evidence of heightened performance on the measure of cognitive flexibility. However, there were no associations between EF performance and measures of caregiver enrichment, SES or caregiver education in either site except an association between enrichment activities and working memory in the Gambian sample.

 McCann et al., 2023

 Iron status in early infancy is associated with cognitive development up to pre-school age in rural Gambia.

 Iron status in early infancy and trajectories of cognitice development using MSEL and the Eye-Tracking Gap Overlap task.

 Iron status ( measured by soluble transferrin receptor -sTfR) at 5 months of age is associated with MSEL cognitive score at 5 months and trajectory of cognitive development from 5months to 5 years. sTfR at 5 months was also associated with an eye tracking measure of visual attention in cross sectional analysis, however this relationship was diminished by 5 years of age.

 Katus*, Crespo-Llado* et al., 2024

 It takes a village: caregiver diversity and language contingency in the UK and rural Gambia

 LENA data at 12, 18 and 24 months for UK and Gambia.

 In the Gambia, earlier LENA measures of turn taking were associated with subsequent child vocalisations. In the UK, there was a trend toward such associations. In the Gambian cohort, we explored the role of caregiving: variability in caregiver numbers from one day to the next was associated with less turn taking. Total number of caregivers showed an inverted u-shaped relationship with turn taking: a medium number of caregivers was associated with highest turn taking frequencies.

 Macrae et al., (2024)

 Cognitive control in infancy: attentional predictors using a tablet-based measure

 Tablet-based measure of cognitive skills, attentional disengagement (Gap-overlap task) and general cognitive skills (MSEL) at 5-24 months in the UK.

 Participants showed significant improvements (at 24 months compared to 18 months) in cognitive control. General cognitive skills (measured using the MSEL) were not significantly associated with cognitive control, while attentional disengagement at 8 and 18 months was a significant predictor.

 Pilot phase of the BRIGHT Project (2013 – 2014)

 Milosavljevic et al., 2019

**Adaptation of the Mullen Scales of Early Learning for use among infants aged 5- to 24-months in rural Gambia.**

 Pilot data collected using the MSEL, and measures of physical growth (height-for-age [HAZ], weight-for-height [WHZ], Head Circumference [HCZ]), collected at 9-24 months in The Gambia.

 Primarily, we report on the method adapting the MSEL for use in the rural Gambian context. We also find that infants show normative MSEL performance early in infancy (at 5-9 months), however from 10-14 months, they show poorer performance compared to normed scores. Additionally, both HAZ and WHZ were significantly associated with MSEL performance.

 Lloyd-Fox et al., (2017)

**Cortical specialisation to social stimuli from the first days to the second year of life: A rural Gambian cohort.**

 Pilot fNIRS and anthropometric data collected in first wave of longitudinal and cross-sectional analyses of infants aged 1 – 24 months.

 Lloyd-Fox et al., (2014)

**Functional Near Infrared Spectroscopy (fNIRS) to assess cognitive function in infants in rural Africa.**

 Pilot fNIRS data collected during first study in The Gambia – 4-8 month olds watched videos of a auditory and visual social cognition paradigm; a task showing Gambian adults performing social movements (i.e. Peekaboo) or vocalisations vs non-social stimuli

 Distinct regions of the posterior superior temporal and inferior frontal cortex evidenced either visual-social activation or vocally selective activation (vocal > non-vocal). The patterns replicated those observed within similar aged infants in previous studies in the UK. These are the first reported data on the measurement of localized functional brain activity in young infants in Africa

 Begus et al., (2016)

**Using fNIRS to study working memory in infants in rural Africa.**

 Pilot fNIRS data collected in cross-sectional analyses - 12-16 month olds watched videos of an *object permanence *paradigm; a task testing the ability to create and hold a mental schema of an object in mind, when it is no longer visually accessible

 Differential neural activity was seen when infants observed objects being hidden for 3 (posterior superior temporal activity) compared to 6 seconds (additional regions of inferior frontal and anterior superior temporal activity). This cortical activation could potentially reflect working memory and executive functioning.

 Papademetriou et al, (2014)

**Optical imaging for brain activation in Gambian infants.**

 Optical topography of fNIRS data from Lloyd-Fox et al., 2014

 A finite element model to model the propagation of light through tissue and create optical topography images of brain activation

 Blasi et al., (2014)

 Test–retest reliability of functional near infrared spectroscopy in infants

 Test re-test reliability of fNIRS signals from longitudinal data collection at 4 – 8 and 12 – 16 months of age

 At the group level, good spatial overlap of significant responses and signal reliability was seen At participant level, spatial overlap was acceptable although results highlighted that signal reliability varied between participants.

# Reviewer report: Reviewer response for version 1

**DOI:** 10.21956/gatesopenres.16121.r35400

**Published:** 2023-11-20

**Authors:** Prerna Aneja

**Affiliations:** 1 University of East Anglia, Norwich, England, UK

The article makes an important contribution by highlighting the need to understand developmental trajectories in context of adversity. The paper proposes a comprehensive project through a longitudinal design and by considering various socio-cultural (risk and protective) factors. The key aims of the research include developing brain and neurocognitive function-for-age curves, establishing associations between poverty-related risk factors and developmental trajectories, and examining associations between context variables and preschool outcomes (long term outcomes). To this end, the authors outline the project design to investigate early brain and cognitive development in both The Gambia and UK (with the former being the primary focus of the project) as well as the developmental outcomes at 3-5 years of age in The Gambia.

**Methods:**

**NBAS:**

 The authors mention introducing controversial items with “special care”. A short follow up with some concrete information could help the reader understand better what the special care would entail for work with this community. The authors mention that some aspects of task have been negatively perceived in other countries (I interpret that this falls outside of their own pilot). A citation for the research carried out in other nations would be helpful for readers who would like to do further readings on evaluations of the NBAS. Last, given the statement of “other countries” is not very concrete (e.g., do “other countries” include western nations?), it was not clear if added care was also necessary when using the clinical assessment in the UK. Clarifying this point would be helpful.

**Parent-child interaction videos:**

 The authors provide a good and clear general overview of the value and function of addressing PCI. To further enhance clarity, it would be helpful if the authors elaborated on points such as “videos can be coded to assess multiple aspects of parental and child behaviour and engagement”. Are there specific key measures that the authors are planning to focus on or whether they are taking a more open and exploratory approach?

**Sleep Diary:**

 The Sleep diary section mentions that the diary was provided to caregivers. However, given the variability in caregiving figures across ages (as noted in the demographic characteristics section), I wondered whether the sleep diary was initially provided to mothers specifically (due to potentially being expected to act as primary caregivers), to other family members (e.g., grandparents), or if it was shared with the whole family for anyone to complete. Similarly, I wondered whether the person completing the sleep journal changed over time, given that secondary and tertiary caregivers shifted as children grew older.

**PANAS:**

 The authors note that some of the English words used in the PANAS were not differentiable in Mandinka, so the number of items were reduced in The Gambian version relative to the one administered in the UK. What was the total number of items for the positive and negative affect scores in the Gambia PANAS measure? It also may be worth it to address some information about which specific positive and negative affect words had to be reduced. For instance, the negative affect in the PANAS includes measures linked to emotions of fear (e.g., afraid and scared) as well as items conceptually linked to anger (e.g., hostile). Given that the emotions have some distinct characteristics (despite being grouped as negative), it could be helpful for readers to understand which specific dimensions of negative affect were affected most by the loss of items (e.g., did all items linked to a specific emotion, let’s say feelings of anger, get removed from the scale? Did all emotional dimensions lose some items but keep others?).

**Results:**

**BRIGHT Kids & Motor Skills:**

 The authors use an adaptation of the TGMD. It may be useful for the authors to draw from research that shows food reliability and consistency of the scale across a few countries to support their use of the measure (e.g., Rey *et al.*, 2020 ^1^ ).

**Parent-report measures:**

 Given the nuanced approach to cultural and contextual sensitivity in the project and considering the complex influences that the COVID pandemic exerted across different communities, I wondered whether the Impact of COVID-19 was completed as a questionnaire by family members on their own, through an interview (e.g., by a field assistant, or through telephone calls, as noted in regards to the dietary data). Adding a bit more concrete information on the measure (e.g., example of items or scales options for responses as done in the Parental Mental Health section) would be helpful.

Is the study design appropriate for the research question?

Yes

Is the rationale for, and objectives of, the study clearly described?

Yes

Are sufficient details of the methods provided to allow replication by others?

Partly

Are the datasets clearly presented in a useable and accessible format?

Yes

Reviewer Expertise:

Infant cognitive development, visual attention, cultural

I confirm that I have read this submission and believe that I have an appropriate level of expertise to confirm that it is of an acceptable scientific standard, however I have significant reservations, as outlined above.

## References

[1] : Reliability of the test of gross motor development: A systematic review. *PLoS One* .2020;15(7) : 10.1371/journal.pone.0236070 e0236070 10.1371/journal.pone.0236070 32673358 PMC7365594

# Response

**Published:** 2024-05-12

**Authors:** Sarah Lloyd-Fox

We thank you for your helpful and constructive comments. We have addressed all comments in our revision and believe this has strengthened the manuscript. Below we provide detailed responses to the individual points raised (your points in bold, ours in standard or italicised. 

**The article makes an important contribution by highlighting the need to understand developmental trajectories in context of adversity. The paper proposes a comprehensive project through a longitudinal design and by considering various socio-cultural (risk and protective) factors. The key aims of the research include developing brain and neurocognitive function-for- age curves, establishing associations between poverty-related risk factors and developmental trajectories, and examining associations between context variables and preschool outcomes (long term outcomes). To this end, the authors outline the project design to investigate early brain and cognitive development in both The Gambia and UK (with the former being the primary focus of the project) as well as the developmental outcomes at 3-5 years of age in The Gambia.**

**Methods:**

1. **NBAS:**

**The authors mention introducing controversial items with “special care”. A short follow up with some concrete information could help the reader understand better what the special care would entail for work with this community. The authors mention that some aspects of task have been negatively perceived in other countries (I interpret that this falls outside of their own pilot). A citation for the research carried out in other nations would be helpful for readers who would like to do further readings on evaluations of the NBAS. Last, given the statement of “other countries” is not very concrete (e.g., do “other countries” include western nations?), it was not clear if added care was also necessary when using the clinical assessment in the UK. Clarifying this point would be helpful.**

**Response: **We agree that this added information would be useful to the reader, and have thus provided the below information:

*In line with feedback from parents from other countries (across low-, middle- and high-income settings, see Katus et al., 20204) and populations, a few aspects of the assessment (specifically, shining a light over closed eyes while sleeping, covering eyes with cloth while awake and undressing the infant) were questioned or perceived negatively (Super et al., 2020). While no items were altered or removed from the assessment, the more controversial items were introduced with special care during all study visits.* *Adjustment to the protocol included to either i) provide additional explanation on the rationale for an item (e.g., for habituation to light, or covering the infant’s eyes) to check whether parents approved of the item’s administration (e.g., undressing); or ii) allow examiners to skip an item if faced with an unsuitable environment (e.g., no suitable surface to assess reflexes). Overall, the interviews emphasised the need to communicate with caregivers prior to and during the assessment, and in addition to observe the infant and be aware of cues from parents throughout. Another recurrent theme was that parents allowed certain actions to be performed specifically because they were part of a research study. This emphasised the ethical responsibility of making sure all items administered were relevant and provided a benefit to the participant or the wider community. In both the UK and The Gambia, the NBAS was performed between 7-14 days after birth during a pre-arranged home visit. Administration times for the NBAS ranged from 20 to 45 minutes, depending on the infants’ state of alertness.*

1. **Parent-child interaction videos:**

**The authors provide a good and clear general overview of the value and function of addressing PCI. To further enhance clarity, it would be helpful if the authors elaborated on points such as “videos can be coded to assess multiple aspects of parental and child behaviour and engagement”. Are there specific key measures that the authors are planning to focus on or whether they are taking a more open and exploratory approach?**

**Response: **Thank you for this suggestion, the following has been added to the main text to address this: 

*Coding parent-infant interactive styles is an ongoing process, with different approaches undertaken at different age points. At the 1- and 5-month visits, we implemented an adapted version of the Global Rating Scales (Murray et al., 1996) that had previously been used with rural and/or low-income communities in South Africa and India (Bozicevic et al., 2016; Cooper et al., 2002; Holla et al., 2021; Tomlinson et al., 2005). The GRS assesses maternal (contingency, responsiveness, affect), infant (active communication, attentiveness) and interactive (level and intensity of dyadic engagement) characteristics. The GRS have been designed to be comparable across cultures by ensuring that key dimensions are assessed independently of modality. Thus, the main adaptation involved removing items that relied heavily on speech content, as prior work demonstrated that, at this young age, speech content does not influence infant behaviour (Bozicevic et al., 2016; Stern et al., 2016). However, as infants get older, both language and cultural norms play an increasing role in social interactions. Thus, it is our aim to develop objective, culturally tailored assessments, of parent-infant interactions, rather than relying solely on schemes developed for other cultures. Our approach in developing these new coding schemes thus far have been to run focus groups with Gambian research staff. These involve an iterative process whereby we begin by asking for reflections on caregiving and infant/child behaviours (across different ages) within this population; then ask open questions within the group while viewing videos to describe behaviours seen and whether they are frequently used by families living in this cultural setting; then introduce the group to commonly used coding schemes; and finally co-create a coding scheme that combines the advantages of standardised frameworks with the specificity required for a given context.   *

1. **Sleep Diary:**

**The Sleep diary section mentions that the diary was provided to caregivers. However, given the variability in caregiving figures across ages (as noted in the demographic characteristics section), I wondered whether the sleep diary was initially provided to mothers specifically (due to potentially being expected to act as primary caregivers), to other family members (e.g., grandparents), or if it was shared with the whole family for anyone to complete. Similarly, I wondered whether the person completing the sleep journal changed over time, given that secondary and tertiary caregivers shifted as children grew older.**

**Response: **We have added the following clarification to the section describing the sleep diary to address this point:

*As was the case with the maternal mental health questionnaires, the sleep diary was administered as an interview by field staff with the primary caregiver who accompanied the child to the visit. This was most commonly the mother across study visits.*

1. **PANAS:**

**The authors note that some of the English words used in the PANAS were not differentiable in**

**Mandinka, so the number of items were reduced in The Gambian version relative to the one**

**administered in the UK. What was the total number of items for the positive and negative affect scores in the Gambia PANAS measure? It also may be worth it to address some information about which specific positive and negative affect words had to be reduced. For instance, the negative affect in the PANAS includes measures linked to emotions of fear (e.g., afraid and scared) as well as items conceptually linked to anger (e.g., hostile). Given that the emotions have some distinct characteristics (despite being grouped as negative), it could be helpful for readers to understand which specific dimensions of negative affect were affected most by the loss of items (e.g., did all items linked to a specific emotion, let’s say feelings of anger, get removed from the scale? Did all emotional dimensions lose some items but keep others?).**

**Response: **We have added the following to the description of the PANAS to address this query:

*Furthermore, some of the English words used, in the PANAS, were not differentiable in Mandinka, and so the number of items were reduced in The Gambian version relative to the one administered in the UK. The Mandinka version of the PANAS included 6 items relating to PA (Curious, Happy, Strong, Proud, Determined, Active) and 6 items relating to NA (Distressed, Regretful, Scared, Aggressive, Ashamed, Uncomfortable).*

**Results:**

1. **BRIGHT Kids & Motor Skills:**

**The authors use an adaptation of the TGMD. It may be useful for the authors to draw from**

**research that shows food reliability and consistency of the scale across a few countries to support their use of the measure (e.g., Rey et al., 20201).**

**Response: **We thank the reviewer for this comment and suggested reference, we include the below in the main text to address this:

 The TGMD (in its various editions) has been used to assess GM skills in multiple countries with good reliability and consistency across settings (Rey et al., 2020). Of note is that it has successfully been implemented in other rural African communities to assess pre-school and school-aged children (Cook et al., 2019; Tomaz et al., 2019; Tsikata et al., 2021).

1. **Parent-report measures:**

**Given the nuanced approach to cultural and contextual sensitivity in the project and considering the complex influences that the COVID pandemic exerted across different communities, I wondered whether the Impact of COVID-19 was completed as a questionnaire by family members on their own, through an interview (e.g., by a field assistant, or through telephone calls, as noted in regards to the dietary data). Adding a bit more concrete information on the measure (e.g., example of items or scales options for responses as done in the Parental Mental Health section) would be helpful.**

**Response: **We have included the below in the description of the Covid-19 questionnaire to address this.

*Impact of COVID-19*

*The BRIGHT Kids follow up was conducted during the COVID-19 pandemic and, thus, it was vital to better understand how the pandemic impacted on each family’s wellbeing, financial situation and access to healthcare. The questionnaire was adapted from a measure that was being used in a UK study at the time to assess the impact of the pandemic on families with young children (Aydin et al., 2022). Items that were deemed by field staff to be irrelevant or unsuitable for the West Kiang community were removed. The final measure consisted of a series of questions that asked the caregiver to report on how their living and financial situation had changed due to the pandemic (e.g., “Has COVID-19 had an impact on your ability to get enough food for your household?”), whether they had family members who got sick with COVID-19 (e.g., “Do you think someone in your household has had, or currently has COVID-19?”), their ability to access healthcare (e.g., “Has COVID-19 impacted your ability to see a doctor or go to the hospital when you or a family member needed to?”), and their general feelings and concerns related to the situation (e.g., “I am afraid of COVID-19” – not at all, a little, a lot). As was the case with the maternal mental health questionnaires, the questionnaire was administered as an interview by field staff with the primary caregiver who accompanied the child to the visit.*

 Once again, we thank the two reviewers for their thorough, thoughtful, and constructive feedback, which will undoubtedly strengthen this manuscript. We hope that we have addressed in sufficient detail.
